# Modulating the Strength and Threshold of NOTCH Oncogenic Signals by *mir-181a-1/b-1*

**DOI:** 10.1371/journal.pgen.1002855

**Published:** 2012-08-09

**Authors:** Rita Fragoso, Tin Mao, Song Wang, Steven Schaffert, Xue Gong, Sibiao Yue, Richard Luong, Hyeyoung Min, Yumi Yashiro-Ohtani, Mark Davis, Warren Pear, Chang-Zheng Chen

**Affiliations:** 1 Department of Microbiology and Immunology, Stanford University School of Medicine, Stanford, California, United States of America; 2 Baxter Laboratory for Stem Cell Biology, Stanford University School of Medicine, Stanford, California, United States of America; 3 Department of Comparative Medicine, Stanford University School of Medicine, Stanford, California, United States of America; 4 Chung-Ang University College of Pharmacy, Seoul, Korea; 5 Abramson Family Cancer Research Institute, University of Pennsylvania School of Medicine, Philadelphia, Pennsylvania, United States of America; 6 Department of Pathology and Laboratory Medicine, University of Pennsylvania School of Medicine, Philadelphia, Pennsylvania, United States of America; 7 Howard Hughes Medical Institute and Department of Microbiology and Immunology, Stanford University School of Medicine, Stanford, California, United States of America; Cincinnati Children's Hospital Medical Center, United States of America

## Abstract

Oncogenes, which are essential for tumor initiation, development, and maintenance, are valuable targets for cancer therapy. However, it remains a challenge to effectively inhibit oncogene activity by targeting their downstream pathways without causing significant toxicity to normal tissues. Here we show that deletion of *mir-181a-1/b-1* expression inhibits the development of *Notch1* oncogene-induced T cell acute lymphoblastic leukemia (T-ALL). *mir-181a-1/b-1* controls the strength and threshold of Notch activity in tumorigenesis in part by dampening multiple negative feedback regulators downstream of NOTCH and pre-T cell receptor (TCR) signaling pathways. Importantly, although Notch oncogenes utilize normal thymic progenitor cell genetic programs for tumor transformation, comparative analyses of *mir-181a-1/b-1* function in normal thymocyte and tumor development demonstrate that *mir-181a-1/b-1* can be specifically targeted to inhibit tumor development with little toxicity to normal development. Finally, we demonstrate that *mir-181a-1/b-1*, but not *mir-181a-2b-2* and *mir-181-c/d*, controls the development of normal thymic T cells and leukemia cells. Together, these results illustrate that NOTCH oncogene activity in tumor development can be selectively inhibited by targeting the molecular networks controlled by *mir-181a-1/b-1*.

## Author Summary

Oncogenes elicit driving signals required for tumor initiation, development, and maintenance and are valuable targets for cancer therapy. However, oncogenes often have essential functions in normal cellular physiology and produce intracellular proteins that are difficult to inhibit with small molecule drugs without causing significant toxicity to normal tissues. Thus, one of the challenges in cancer therapy is to identify downstream networks that can be targeted to specifically dampen the oncogenic signals in tumor cells without harming normal tissues. In this study we demonstrate that deletion of a microRNA (miRNA) gene, *mir-181a-1/b-1*, specifically inhibits the activity of the *Notch* oncogene in tumorigenesis without causing significant defects in normal development. Although earlier studies have elegantly shown the essential role of NOTCH and pre-TCR signals in NOTCH-induced tumorigenesis, neither NOTCH nor pre-TCR signals can be targeted effectively for treatment of T-ALL with available drugs due to their weak therapeutic effects and severe toxicities. Our findings illustrate that dissecting the downstream targets of miRNAs can reveal the molecular networks that can be targeted to control tumor transformation caused by oncogenes. More importantly, our results illustrate that comparative studies on the pathways utilized by normal cells and tumor cells may reveal novel insights into how tumorigenic pathways may be selectively inhibited with limited damage to normal tissues.

## Introduction

Oncogenes elicit driving signals required for tumor initiation, development and maintenance and are valuable targets for cancer therapy. However, oncogenes often have essential functions in normal cellular physiology and produce intracellular proteins that are difficult to inhibit with small molecule drugs without causing significant toxicity to normal tissues. Therefore, it is imperative to identify downstream networks that can be targeted to dampen the oncogenic signals in tumor cells with limited toxicity to normal cells. Despite intense efforts, it remains a challenge to identify the downstream pathways controlled by oncogenes that are essential and specific for tumor development but not for normal development.

MicroRNAs (miRNAs) are an abundant class of small regulatory RNAs that control gene expression at the post-transcriptional levels. Some miRNAs are capable of potentiating tumorigenic activity of oncogenes, such as *Myc* and *Notch1*, possibly by repressing known tumor suppressors [1], [2]. In some cases, miRNAs can function as oncogenes and aberrant expression of such an miRNA is sufficient to induce cancer [3], [4]. Finally, some miRNAs can inhibit activities of oncogenes when delivered into tumor cells through viral transduction [5], [6]. Clearly, miRNAs are integral components of oncogenic and tumor suppressing networks; however, the quantitative nature of miRNA effects on gene expression and cellular functions raises the issue as to whether loss of a miRNA would be sufficient to inhibit oncogene-induced tumorigenesis [7], [8], [9]. More importantly, few studies have demonstrated that miRNAs have essential roles in tumor development caused by human oncogenes using rigorous loss-of-function analyses.

We used a NOTCH-induced T-cell acute lymphoblastic leukemia (T-ALL) model [10] to gain insight into how activity of human oncogenes is modulated by miRNAs. Activating mutations in *Notch1*, which cause ligand-independent activation of the receptor and/or inhibit proteasome-mediated receptor turnover, are observed in about 60% of human T-ALL cases [11]. The requirement of γ-secretase for NOTCH1 activation led to the clinical evaluation of γ-secretase inhibitors (GSIs) for the treatment of T-ALL. Treatment of patients with these inhibitors was unsuccessful because of limited anti-leukemic activity and severe gastrointestinal toxicity [12]. In humans and in animal models, transformation by *Notch1* oncogenes blocks T cell development at the immature double-positive (DP) cell stage but not at the mature T cell stages [13], indicating that *Notch1* oncogenes may utilize the genetic programs that operate in normal thymic progenitor cells for tumor transformation. Intriguingly, miR-181a family miRNAs are highly expressed in T-ALL leukemia cells and down-regulated during remission [14], suggesting that miR-181 miRNAs play a role in the pathogenesis of human T-ALL. Here we carried out comparative analyses to examine the roles of *mir-181* alleles in normal development and NOTCH-induced T-ALL using a loss of function approach. We found that *mir-181ab1* controls the strength and threshold of *Notch1* oncogenes by repressing the negative feedback regulators downstream of NOTCH and pre-TCR signaling. Deletion of *mir-181ab1* effectively inhibits NOTCH-induced T-ALL without significant impact on normal development. These findings illustrate a general approach in uncovering pathways that are essential for oncogene activity in tumor development.

## Results

### Targeted deletion of *mir-181* alleles in mice

**Figure 1:**
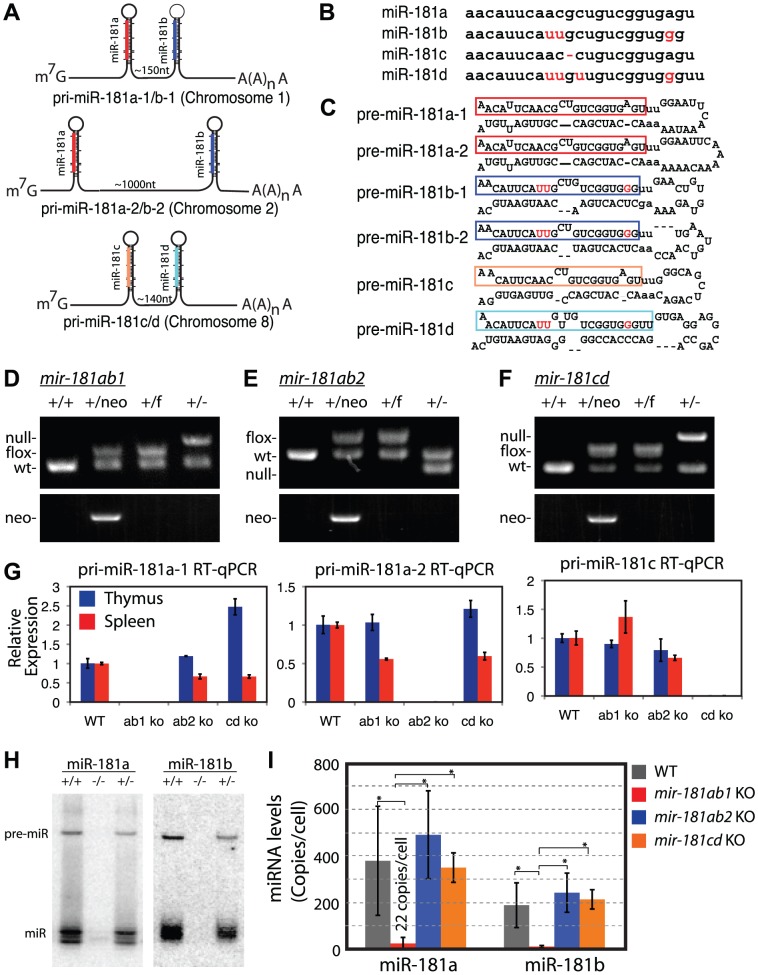
Generation of conditional *mir-181* alleles in mice. (A) Schematics of putative primary miR-181 transcripts. (B) Alignment of mouse miR-181 family mature miRNAs. (C) Predicted stem-loop structures of miR-181 family miRNAs. (D-F) PCR confirmation of the deletion of neo cassette and (D) *mir-181ab1*, (E) *mir-181ab2* and (F) *mir-181c/d*. (G) RT-qPCR analyses of primary miR-181 expression in the thymus (blue) and spleen (red) of *mir-181* knockout mice. (H) Northern blot analyses to confirm loss of miR-181a and miR-181b expression in the thymus upon the loss of *mir-181ab1* alleles. (I) Mature miR-181a and miR-181b expression in DP cells from WT, *mir-181ab1* ^−/−^ and *mir-181ab2* ^−/−^ mice determined by quantitative miRNA PCR analyses (n = 2, *, *p*<0.05, Student's *t*-test).

The members of the miR-181 family of genes, which will be referred to as *mir-181ab1*, *mir-181ab2* and *mir-181cd*, produce four nearly identical mature miRNAs (miR-181a, miR-181b, miR-181c and miR-181d, respectively) from three polycistronic transcripts (Figure 1A–C). Given the dynamic expression of miR-181 miRNAs during normal lymphocyte development and during T-ALL progression [14]–[18] and that *Notch1* oncogenes utilize genetic programs in early thymic progenitor cells for T-ALL development [13], we used loss-of-function analyses to identify the *mir-181* allele with critical roles in the development of normal thymic progenitor cells and in development of NOTCH-induced T-ALL. We obtained conditional mouse strains for all *mir-181* alleles (Figure 1D–F and Figure S1A–G). Germline deletion of each individual *mir-181* allele completely abolished expression of pri-miRNA transcripts from the corresponding allele (Figure 1G). No protein coding genes were found within 10 kb up- or downstream of the *mir-181ab1* and *mir-181ab2* alleles (Figure S1G), and it is unlikely that loss of *mir-181ab1* and *mir-181ab2* would affect expression of protein-coding genes further from the alleles. Interestingly, *Nanos3*, a germline specific gene that plays an essential role in germ cell development, is about 2 kb upstream of *mir-181cd* (Figure S1G). However, *mir-181cd* null mice are viable and have no apparent defects in fertility. Thus, loss of *mir-181cd* did not affect *Nanos3* expression to a degree that would compromise the fertility of *mir-181cd* null mice.

Since *Notch1* oncogenes may utilize the genetic programs that operate in normal thymic progenitor cells for tumor transformation [13], we further characterized mature miR-181 miRNA expression in these germline knockout mice to determine whether one or more alleles contribute to miR-181 expression in thymocytes. T cell differentiation in the thymus can be divided into discrete stages based on CD4 and CD8 expression: CD4 and CD8 double-negative (DN) early thymic progenitors, more differentiated CD4 and CD8 double-positive (DP) thymocytes and differentiated CD4 or CD8 single-positive (SP) thymocytes. DN cells are further subdivided into DN1 (CD44^+^ CD25^−^), DN2 (CD44^+^ CD25^+^), DN3 (CD44^−^ CD25^+^) and DN4 (CD44^−^ CD25^−^) cell populations, listed in the order of their appearance during development. The DN1 subset also encompasses the earliest thymic T-cell progenitors, ETPs [19]. Previous sequencing analyses of small RNAs in CD4 and CD8 DP cells showed that mature miR-181a is expressed at levels about 100-fold higher than those of mature miR-181c [17]. Interestingly, loss of the *mir-181ab1* allele reduced mature miR-181a expression to near background levels in the thymus as indicated by the northern blot analyses (Figure 1H). Furthermore, as shown by qPCR analyses, loss of the *mir-181ab1* allele reduced mature miR-181a and mature miR-181b expression to ∼22 and 8 copies per cell in DP cells (Figure 1I), respectively. Deletion of *mir-181ab2* and *mir-181cd* alleles did not affect mature miR-181a and miR-181b expression levels (Figure 1I). Together, these results demonstrate that miR-181a and miR-181b are predominantly expressed from *mir-181ab1* in thymocytes, suggesting that *mir-181ab1*, but not *mir-181ab2* and *mir-181cd*, may play a specific role in early thymocyte development.

### *mir-181ab1*, but not *mir-181ab2* or *mir-181cd*, affects early thymocyte development

**Figure 2:**
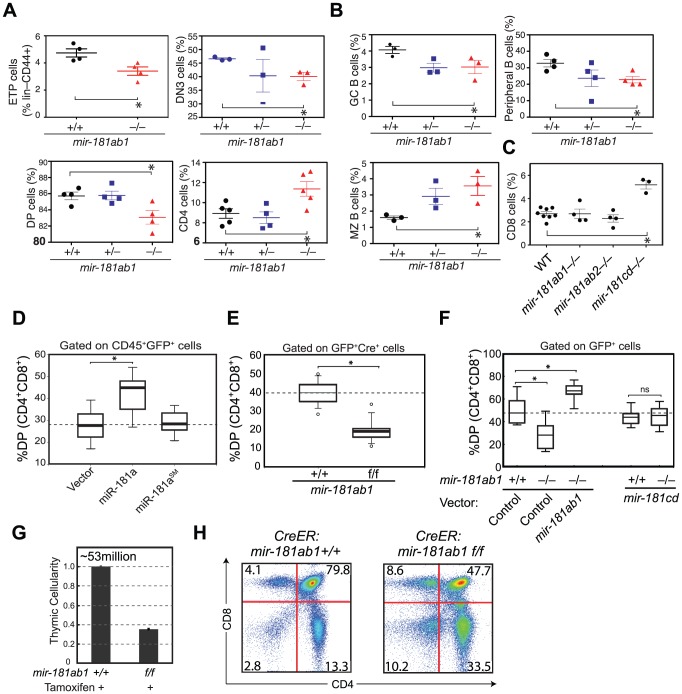
Effects of *mir-181ab1* deletion on normal development. (A and B) Effects of germline deletion of *mir-181ab1* on (A) T lymphocyte and (B) B lymphocyte development (*, *p*<0.05, Mann-Whitney rank sum tests). (C) Effects of germline deletion of *mir-181cd* on CD8 thymocyte development. (D) Effects of *mir-181a-1* ectopic expression on DP thymocyte development determined by the OP9-DL1 assay. (E) Effects of Cre-mediated deletion of *mir-181ab1* on DP thymocyte development determined using the OP9-DL1 assay. (F) Effects of germline *mir-181ab1* deletion and *mir-181cd* deletion on DP thymocyte development in the OP9-DL1 assay. (G and H) Effects of induced deletion of the *mir-181ab1* alleles on thymocyte development *in vivo*. CreER:*mir-181ab1^+/+^* and CreER:*mir-181ab1^f/f^* mice were injected with tamoxifen four times at two-day intervals to induce the deletion of *mir-181ab1* alleles and analyzed at day 5 after the last tamoxifen injection to determine (G) thymic cellularity and (H) CD4/CD8 profiles. Representative results of two independent analyses are summarized or presented in (G) and (H).

We then systematically examined the consequences of loss of individual *mir-181* alleles on the development of T and B lymphocyte populations in various lymphoid and hematopoietic organs with a focus on the thymocyte populations (see Table S1 for cell populations and FACS definitions of ∼40 individual T and B lymphocyte populations). Consistent with predominant expression of mature miR-181a and miR-181b from *mir-181ab1*, the deletion of *mir-181ab1* caused more apparent defects in early thymocyte development than deletion of the other alleles. We noted statistically significant but modest decreases in ETP, DN3 and DP cell populations and an increase in CD4 SP thymocytes upon *mir-181ab1* deletion (Figure 2A and Figure S2A, B). *mir-181ab1* deletion also resulted in significant changes in germinal center, marginal zone and peripheral B cell development (Figure 2B). Germline deletion of *mir-181cd* caused more than a 2-fold increase in the percent of CD8 T cells in the thymus but did not cause notable changes in other T and B lymphocyte populations examined including DN1–4, DP and CD4 cells (Figure 2C). Germline deletion of *mir-181ab2* had no apparent effects on any of the cell populations examined. Cell populations without significant changes upon the loss of individual *mir-181* alleles are not shown in Figure 2. Moreover, loss of individual *mir-181* alleles did not significantly change the cellularity of hematopoietic organs, such as bone marrow, spleen, thymus and peripheral blood, in the germline-knockout mice. Thus, changes in proportions shown in Figures 2A–C should correlate with corresponding changes in cell numbers. miR-181 miRNAs are also expressed in the brain and in muscle [15], but further analyses must be carried out to dissect the function of *mir-181* alleles in these tissues. Overall, germline *mir-181* knockout mice are viable and have no noticeable defects for up to twelve months.

The effects of *mir-181ab1* on early T cell development were further confirmed with *in vitro* and *in vivo* analyses. Using an OP9-DL1 stromal (expressing NOTCH ligand, delta-like 1) co-culture assay [20] that recapitulates early thymic T cell development in culture (Figure S2C), we showed that ectopic *mir-181a-1* expression in thymic progenitor cells potentiates DP cell development (Figure 2D, and Figure S2D,E), whereas conditional (Figure 2E and Figure S2F–I) or germline (Figure 2F) deletion of *mir-181ab1* inhibits it. The ectopic expression of *mir-181ab1*, but not *mir-181cd*, in thymic progenitors rescued the defects caused by *mir-181ab1* germline deletion (Figure 2F), demonstrating that these two alleles have different functions in early T cell development [21]. Most importantly, conditional deletion of the allele using tamoxifen-induced CreER resulted in 50–75% decrease in cellularity in the thymus (Figure 2G, *p*<0.05) – a decrease from an average of ∼53 million cells/thymus in wild-type mice to ∼23 million cells/thymus in mice with *mir-181ab1* alleles floxed – and a significant reduction in the percentage of DP cells (Figure 2H, *p*<0.05). Finally, miR-181a expression decreased during the DN3a to DN3b transition during β-selection (Figure S2J), and loss of *mir-181ab1* resulted in a significant reduction in the percentage of DN3 and DN4 cells that expressed intracellular TCR-β (Figure S2K), but preTα expression in DN3 subsets was normal (data not shown). These results suggest that *mir-181ab1* contributes to the DN3 to DP transition, possibly by regulating β-selection and post β-selection expansion, and its effects are intrinsic to thymic progenitor cells. Importantly, the fact that strong effects on thymic T cell development as a result of *mir-181ab1* deletion *in vitro* (Figure 2E–F) and acute deletion of *mir-181ab1 in vivo* were observed (Figure 2G, H), suggests that it is likely that such effects might be compensated in germline knockout mice through homeostatic mechanisms that maintain the stability and robustness of immune systems (Figure 2A–C). In summary, *mir-181ab1*, but not *mir-181ab2* or *mir-181cd*, has specific roles in early T cell development in the thymus.

### Loss of *mir-181ab1* dampens ICN1 oncogenic activity in T-ALL induction

The fact that *Notch1* oncogenes block T cell development at the immature DP cell stage but not at the mature T cell stages [13] suggests that *Notch1* oncogenes utilize the genetic programs of normal thymic progenitor cells for T-ALL induction. Indeed, NOTCH and pre-TCR signals, which act synergistically to promote thymic T cell development during β-selection and post β-selection T cell expansion, are also critical for NOTCH-induced T-ALL development [22], [23]. Given the role of *mir-181ab1* in early thymocyte development and during the DN3 to DP transition (Figure 2D–H and S2J, K), *mir-181ab1* may also be important for NOTCH-induced T-ALL.

**Figure 3:**
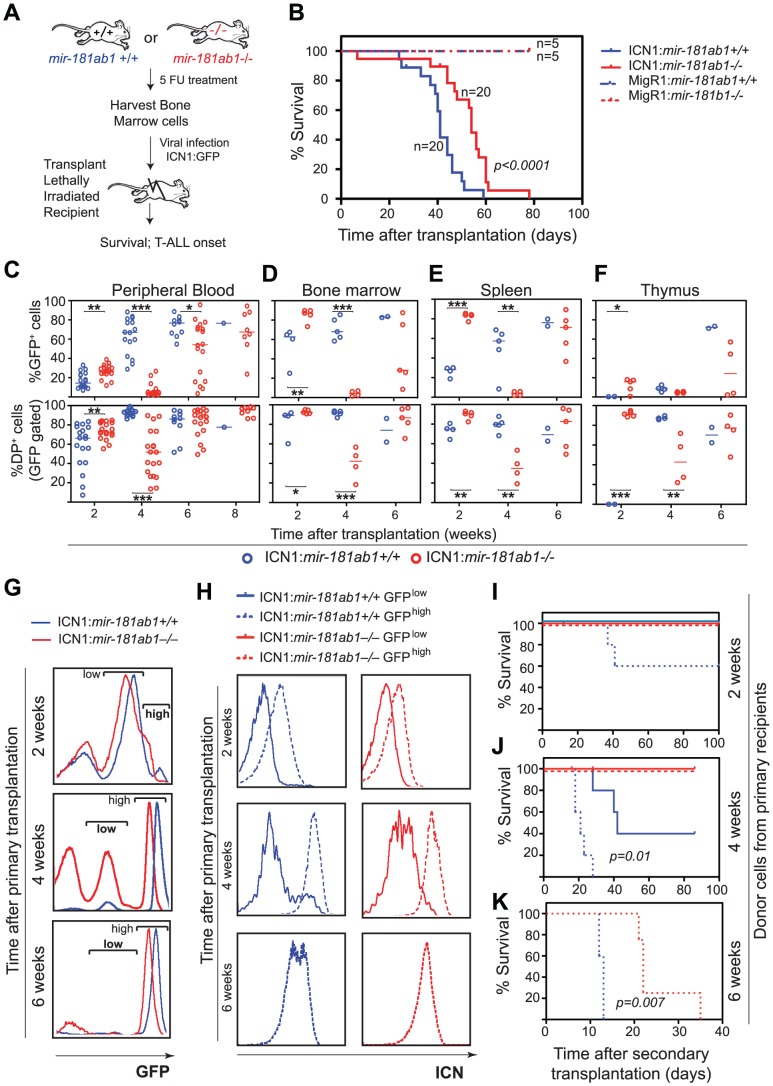
Loss of *mir-181ab1* delays T-ALL and inhibits T-ALL development induced by low levels of ICN1. (A) Schematic of experimental design. (B) Kaplan-Meier survival curves show the effect of loss of *mir-181ab1* on the percentages of mice surviving at different time points after T-ALL induction with ICN1 (*p*<0.0001, n = 20 mice/experimental group, a representative plot of 4 independent experiments is shown). (C–F) Effects of loss of *mir-181ab1* on the percentage of total ICN1-infected cells (all GFP^+^ cells) and the percent of ICN1-infected DP leukemia cells (GFP^+^DP cells) in (C) peripheral blood, (D) bone marrow, (E) spleen and (F) thymus of T-ALL mice (*, *p*<0.05; **, *p*<0.01; ***, *p*<0.001;). (G) Mice transplanted with ICN1:*181ab1^+/+^* or ICN1:*181ab1^−/−^* BM cells have distinct profiles of GFP^+^ cells at 2, 4 and 6 weeks after transplantation. Gates that define GFP^high^ and GFP^low^ cells are indicated. (H) Relative levels of ICN1 expression in GFP^high^ and GFP^low^ cells were determined by intracellular staining of ICN1 and FACS analyses. (I–K) The Kaplan-Meier survival curves of the secondary transplantation analyses. ICN1-infected DP cells (GFP^+^ DP cells) were sorted from primary recipients at (I) 2, (J) 4 and (K) 6 weeks after transplantation and transferred to each of five secondary recipients. Secondary recipients were analyzed for survival and development of DP leukemia cells in the peripheral blood to determine the leukemogenic potentials of GFP^high^ and GFP^low^ cells from primary recipients transplanted with ICN1:*181ab1^+/+^* and ICN1:*181ab1^−/−^* BM cells.

We next examined the effects of loss of *mir-181ab1* on T-ALL induced by the intracellular domain of NOTCH1 (ICN1) (Figure 3A). Loss of *mir-181ab1* caused a 32% increase in the median survival time of T-ALL mice from 41 days to 54 days (Figure 3B, *p*<0.0001, 20 mice/group, a representative plot of 4 independent experiments is shown). The delayed mortality correlated strongly with a drastic decrease of ICN1-infected blood cells (as measured by the number of GFP^+^ cells) and DP leukemia cells (GFP^+^DP) in the peripheral blood (PB) and in hematopoietic and non-hematopoietic organs of recipient mice at 4 weeks after transplantation (Figure 3C–F and Figure S3A–D). It is important to note the distinct developmental kinetics of GFP^+^ and GFP^+^DP cells in the wild-type and knockout groups. In mice reconstituted with ICN1:*181ab1* ^+/+^ bone marrow (BM) cells, the percentage of GFP^+^ and GFP^+^DP cells in peripheral blood increased during the 6 weeks after transplantation (Figure 3C and Figure S3A), whereas in mice reconstituted with ICN1:*181ab1* ^−/−^ BM cells the percentage of GFP^+^ and GFP^+^DP cells first decreased from 2 to 4 weeks and then increased from 4 to 8 weeks post-transplantation (Figure 3C and Figure S3A).

Loss of *mir-181ab1* also compromised other steps that are required for the development of ICN1-induced T-ALL. For example, ICN1:*mir-181ab1^−/−^* DP cells can develop in the thymus (Figure 3F, bottom panel, 2-week time point), whereas ICN1:*mir-181ab1^+/+^* DP cells cannot. Thus, loss of *mir-181ab1* altered the tissue distribution of ICN1-infected T-ALL cells and enabled the development (or migration) of ICN1-infected DP cells in the thymus at 2 weeks post-transplantation (Figure 3F, lower panel). This is significant because ICN1 expression in hematopoietic progenitor cells is known to block T cell development in the thymus while promoting ectopic T-cell development in BM and other organs [13], [24]. Interestingly, loss of *mir-181ab2* did not inhibit development of ICN1-induced T-ALL, and loss of *mir-181cd* actually exacerbated ICN1-induced T-ALL (Figure S3F–G). Finally, loss of *mir-181ab1* did not have detrimental effects on the reconstitution potentials of hematopoietic stem/progenitor cells (Figure S3E and see Text S1 for additional details and Figure S3H for histology). Since multiple independent infections and large cohorts of recipients were used in each of these analyses, it is unlikely that the effects observed here are due to variations in clonal outgrowth. Together, these results demonstrate that *mir-181ab1*, but not *mir-181ab2* or *mir-181cd*, specifically potentiates ICN1-induced T-ALL.

### *mir-181ab1* deletion affects the strength and threshold of ICN1 oncogenic activity

Intriguingly, we noted that *mir-181ab1* deletion appeared to have stronger inhibitory effects on T-ALL cells with lower levels of ICN1 expression and presumably weaker NOTCH oncogenic signals (Figure 3G–K). Two distinct GFP cell populations were found in the BM of T-ALL mice at 2, 4 and 6 weeks after transplantation: One cell population expressed lower levels of GFP (GFP^low^) and the other cell population expressed higher levels of GFP (GFP^high^) (Figure 3G). As shown by FACS analyses of intracellular ICN1 (Figure 3H), GFP^high^ DP cells have higher levels of ICN1 protein than do the GFP^low^ DP cells. To determine the leukemogenic potential of DP cells with different levels of ICN1 expression, we carried out secondary transplantation analyses. We sorted GFP^high^ and GFP^low^ DP cells from primary ICN1:*181ab1* ^+/+^ and ICN1:*181ab1* ^−/−^ T-ALL mice at 2, 4 and 6 weeks post-transplantation and transplanted sorted DP cells into new recipients to generate secondary T-ALL mice. We then monitored the leukemogenic potential of these DP cells.

Consistent with previous observations on the correlation between the signaling strength of various *Notch1* mutants and T-ALL activity [25], we noted that GFP^high^ DP cells, but not the GFP^low^ DP cells, isolated from ICN1:*181ab1* ^+/+^ T-ALL mice 2 weeks after transplantation caused T-ALL in secondary recipients (Figure 3I). We also observed that the GFP^high^ DP cells caused earlier onset and more aggressive T-ALL than the GFP^low^ DP cells isolated 4 weeks after transplantation (Figure 3J). More importantly, DP cells isolated from ICN1:*181ab1* ^−/−^ primary recipients with either high or low GFP expression levels at 2 or 4 weeks post-transplantation did not induce leukemia in the secondary recipients (Figure 3I, J), whereas the equivalent cell populations from ICN1:*181ab1* ^+/+^ primary recipients did. Finally, although the GFP^high^ DP cells from ICN1:*181ab1* ^+/+^ and ICN1:*181ab1* ^−/−^ primary T-ALL mice collected at 6 weeks after transplantation were strongly leukemogenic, *mir-181ab1* deletion significantly reduced the oncogenic activity of ICN1 (Figure 3K). There were essentially no GFP^low^ DP cells in T-ALL mice at 6 weeks after transplantation. Thus, loss of *mir-181ab1* effectively inhibited T-ALL development in recipients of cells with lower levels of ICN1 expression (GFP^low^ DP cells) (Figure 3J) and delayed T-ALL development in recipients of cells with high levels of ICN1 expression (GFP^high^ DP cells) (Figure 3K). These results also demonstrate that *mir-181ab1* deletion has strong intrinsic effects on the development of DP leukemia cells, although we cannot rule out that this miRNA may affect non-T cell types that can dampen NOTCH-induced T-ALL. Most importantly, these findings show that loss of *mir-181ab1* may be more effective in suppressing T-ALL development induced by *Notch1* mutations with lower levels of ICN1 and weaker signaling strength than that induced by mutations with higher levels of ICN1 and stronger signaling strength. These results demonstrate that *mir-181ab1* controls the strength and threshold of ICN1 oncogenic signals. Thus, *mir-181ab1* deletion can delay T-ALL development induced by strong Notch oncogenes (Figure 3B) and blocks T-ALL development induced by weaker oncogenic signals (Figure 3I–K).

### *mir-181ab1* deletion inhibits T-ALL development induced by a human Notch1 mutant

**Figure 4:**
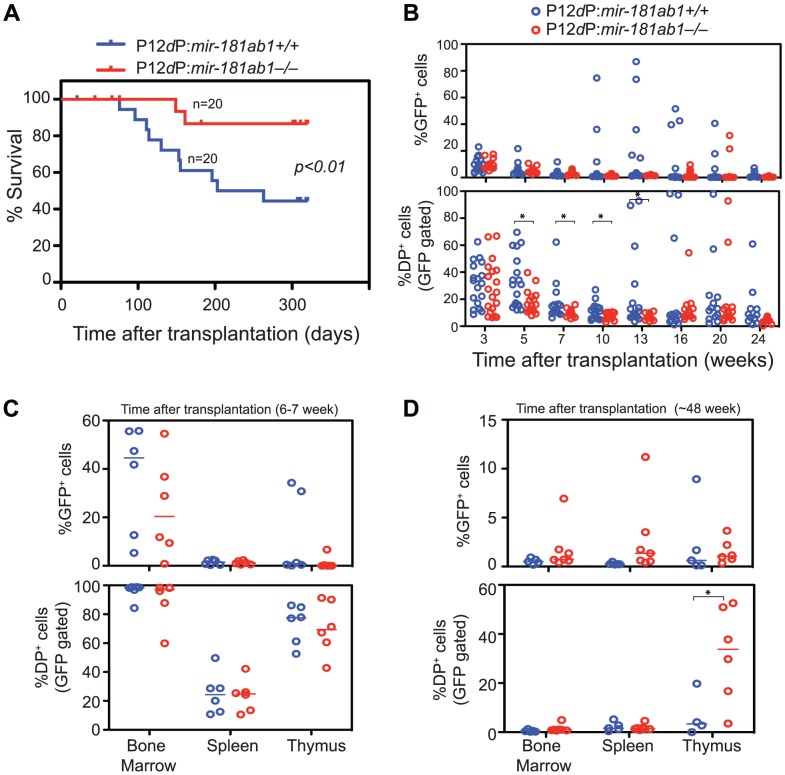
Loss of *mir-181ab1* inhibits leukemia development induced by the human NOTCH1 mutant P12ΔP. (A) Kaplan-Meier survival curve showing the effects of loss of *mir-181ab1* on the percentages of mice surviving at different time points after T-ALL induction with P12ΔP (*p*<0.01, n = 20 mice/experimental group, a representative plot of two independent experiments is shown). (B) Effects of loss of *mir-181ab1* on the percentage of total P12ΔP-infected cells (all GFP^+^ cells) and the percent of P12ΔP-infected DP cells (GFP^+^ DP cells) in peripheral blood at different time points after reconstitution (*, *p*<0.05). (C and D) The percentage of GFP^+^ cells (upper panel) and GFP^+^ DP cells (lower panel) in BM, spleen and thymus of T-ALL mice at (C) 6 to 7 weeks or (D) ∼48 weeks after transplantation (*, *p*<0.05).

Among the known human *Notch1* mutants, the P12ΔP mutant is one of the strongest [25] and is found in 15–20% of pediatric T-ALL patients. Loss of *mir-181ab1* strongly inhibited T-ALL development induced by P12ΔP, causing a decrease in mortality from 60% in P12ΔP:*181ab1^+/+^* T-ALL mice to 10%, a striking 80% reduction in mortality (Figure 4A, 20 mice/group, a representative plot of 2 independent experiments is shown.). Importantly, percentages of P12ΔP-infected PB cells (GFP^+^) and pre-leukemia cells (GFP^+^ DP cells) decreased in both P12ΔP:*181ab1^+/+^* and P12ΔP:*181ab1^−/−^* T-ALL mice at 5 weeks after transplantation (Figure 4B). Infected cells reappeared in 12 out of 20 P12ΔP:*181ab1^+/+^* T-ALL mice (Figure 4B) and caused mortality as early as 10 weeks after transplantation (Figure 4A). In contrast, GFP^+^ DP cells only reappeared in 2 out of 20 P12ΔP:*181ab1^−/−^* T-ALL mice after 20 weeks post-transplantation and caused mortality no earlier than 24 weeks after transplantation. Thus, loss of *mir-181ab1* nearly completely blocked leukemia development induced by P12ΔP. Viral integration effects may have caused higher P12ΔP expression and stronger oncogenic signaling in the two P12ΔP:*181ab1^−/−^* T-ALL mice that died of T-ALL (Figure 4B and Figure S3I).

Comparable percentages of GFP^+^ cells were found in the majority of the hematopoietic/lymphoid organs of P12ΔP:*181ab1^+/+^* and P12ΔP:*181ab1^−/−^* T-ALL mice at 6 to 7 weeks (Figure 4C, upper panel) and at ∼48 weeks after transplantation (Figure 4D, upper panel). Therefore, loss of *mir-181ab1* did not have observable detrimental effects on the reconstitution potential of P12ΔP-infected bone marrow cells. Of note, at ∼48 weeks after transplantation (Figure 4D, lower panel), the P12ΔP:*181ab1^−/−^* T-ALL mice that did not develop leukemia had a significant percentage of P12ΔP-infected (GFP^+^) DP cells in the thymus but not in the PB, BM or spleen (*, *p*<0.05). These results demonstrate that loss of *mir-181ab1* inhibits extrathymic development of P12ΔP-infected (GFP^+^) DP cells and rectifies extrathymic tissue-distribution of oncogenic T-ALL cells previously observed in BM, spleen and other organs [13]. Thus, the loss of *mir-181ab*1 effectively reduced the tumorigenic activity of the P12ΔP oncogene below a functional threshold. These findings further confirm that *mir-181ab*1 deletion effectively inhibits T-ALL development induced by weaker NOTCH oncogenic signals as a result of low ICN1 oncogene expression (Figure 3) or P12ΔP mutations (Figure 4). Since P12ΔP is one of the strongest *Notch1* oncogenes identified in human T-ALL cells, targeting *mir-181ab*1 may effectively inhibit T-ALL development induced by other human *Notch1* mutants.

### *mir-181ab2* and *mir-181cd* do not functionally compensate for the loss of *mir-181ab1* in ICN1-induced T-ALL

**Figure 5:**
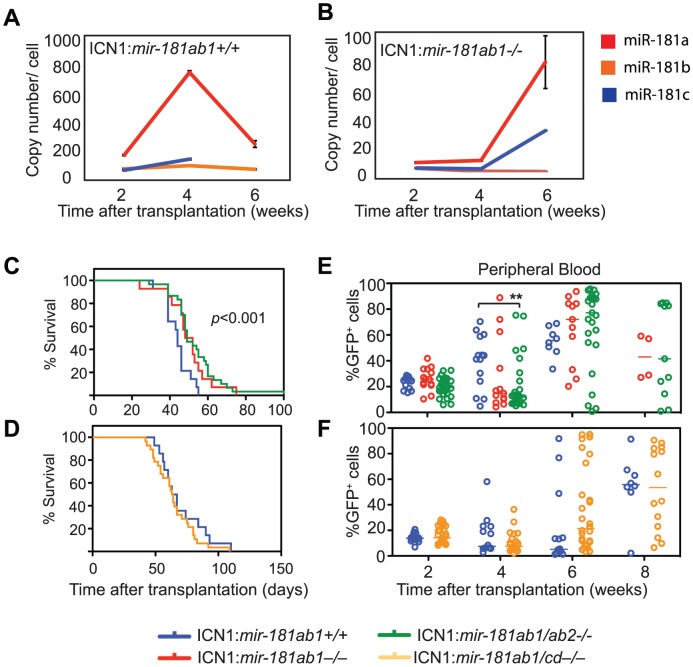
The differential effects of loss of individual *mir-181* family genes on ICN1-induced T-ALL. (A and B) Copy numbers of mature miR-181a, miR-181b, miR181c and miR-181d in ICN1-infected DP cells from (A) ICN1:*181ab1^+/+^* and (B) ICN1:*181ab1^−/−^* recipient mice at 2, 4 and 6 weeks post-transplantation determined by standard curve-based miRNA qPCR analyses. (C and D) Kaplan-Meier survival curves indicate the percentage of mice (20 mice/group) surviving at different time points after reconstituting with (C) ICN1-infected *mir-181ab1/ab2^−/−^* (*p*<0.001) or (D) ICN1-infected *mir-181ab1/cd^−/−^* BM cells (*p*>0.05). (E and F) Effects of loss of *mir-181* family genes on the percentage of total ICN1-infected cells (all GFP^+^ cells) in peripheral blood at different time points after reconstitution (**, *p*<0.01).

The fact that strong NOTCH oncogenic signals can overcome the inhibitory effects of *mir-181ab1* deletion raised the question of whether *mir-181ab2* or *mir-181cd* might compensate for the loss of *mir-181ab1* in T-ALL development. Levels of miR-181a, miR-181b and miR-181c remained at a few copies per cell in ICN1:*181ab1* ^+/+^ T-ALL mice at 2, 4 and 6 weeks after transplantation (Figure 5A), but the levels of miR-181a and miR-181c in ICN1-infected DP cells increased from less than 10 copies/cell at 2 and 4 weeks to 80 and 35 copies/cell, respectively, at 6 weeks post-transplantation in ICN1:*181ab1* ^−/−^ T-ALL mice (Figure 5B). This suggests that ICN1 may up-regulate the expression of miR-181a and miR-181c from *mir-181ab2* and *mir-181cd* alleles, respectively, to compensate for the loss of *mir-181ab1*. Deletion of both *mir-181ab1* and *mir-181ab2* did not further potentiate the effects of loss of *mir-181ab1* on ICN1-induced T-ALL development (Figure 5C, E). In fact, loss of both *mir-181ab1* and *mir-181cd* actually diminished the inhibitory effects of *mir-181ab1* deletion on T-ALL development (Figure 5D, F). Together, these results indicate that increased expression of miR-181a and miR-181c from the corresponding *mir-181ab2* and *mir-181cd* alleles at the late stage of ICN1-induced T-ALL does not compensate for the loss of *mir-181ab1*.

### Loss of *mir-181ab1* effectively dampens ICN1-controlled oncogenic programs

To determine whether *mir-181ab1* affects T-ALL development by directly controlling NOTCH signaling, we carried out transcriptional profiling analyses. To this end we generated triplicate microarray data sets from normal DP thymocytes, primary ICN1:*181ab1* ^+/+^ GFP^low^ DP cells and primary ICN1:*181ab1* ^−/−^ GFP^low^ and GFP^high^ DP cells from T-ALL mice at 4 weeks post-transplantation (Table S2). It is important to note that at 4 weeks post-transplantation, although ICN1:*181ab1* ^−/−^ and ICN1:*181ab1* ^+/+^ DP cells express similar levels of ICN1 (Figure S3J), these cells have different leukemogenic potential. Primary ICN1:*181ab1* ^+/+^ GFP^low^ DP cells induce leukemia in secondary recipient mice, whereas ICN1:*181ab1* ^−/−^ GFP^low^ or GFP^high^ cells do not (Figure 3J). Thus, comparing the changes in gene expression between the primary ICN1:*181ab1* ^+/+^ GFP^low^ DP cells and ICN1:*181ab1* ^−/−^ GFP^low^ or GFP^high^ cells should reveal the effects of *mir-181ab1* deletion on ICN1-controlled oncogenic programs on a global level.

**Figure 6:**
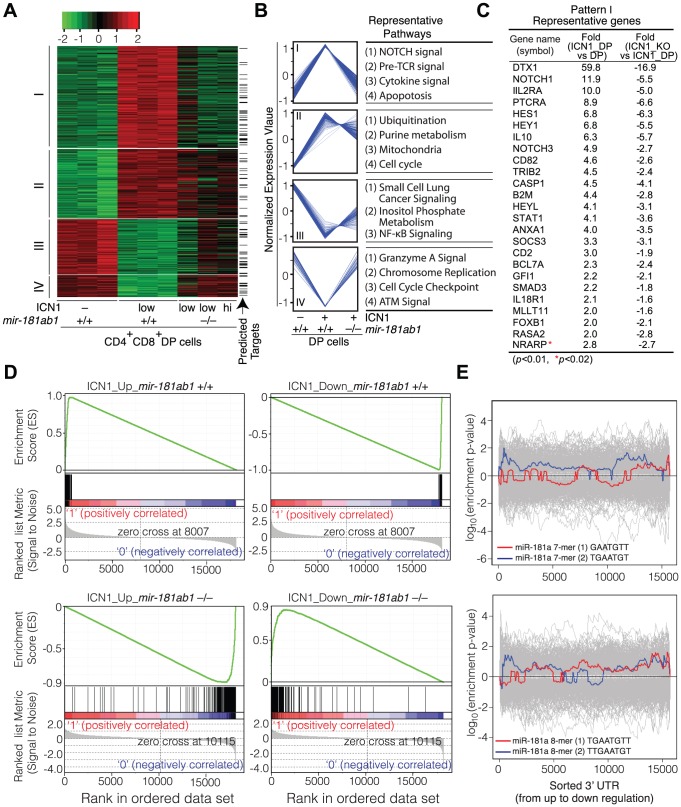
Loss of *mir-181ab1* in DP leukemia cells inhibits ICN1-controlled genetic programs. (A) Hierarchical clustering of genes showing differential expression of ICN1- controlled genes in DP leukemia cells and their expression upon *mir-181ab1* deletion (*p*<0.01 and fold change >2, n = 548). Predicted targets of miR-181a are highlighted and indicated with arrows. (B) The normalized expression patterns of four gene clusters and the representative functional pathways in corresponding clusters (see Table S3 for full list of genes). (C) Representative genes from cluster I and corresponding changes in expression levels in response to ICN1 expression or *mir-181ab1* deletion are shown. (D) Gene set enrichment analyses showing the effects of *mir-181ab1* deletion in the gene sets up-regulated (n = 376; upper left panel) and down-regulated (n = 172; upper right panel) by ICN1. (E) Enrichment of 7- and 8-mer miR-181a seed sequences among the differentially expressed genes caused by the loss of *mir-181ab1* in DP leukemia cells was determined by Sylamer analysis. The colored and gray lines correspond to enrichment of miR-181a seeds and unrelated miRNA seeds, respectively.

ICN1 expression in DP cells resulted in aberrant expression of over 500 genes (>2-fold, *p*<0.01, Table S3). Unsupervised hierarchical clustering analyses classified the ICN1-controlled gene set into four clusters, I–IV (Figure 6A, B). The cluster I genes, which were up-regulated by ICN1 and reverted in the *mir-181ab1* null T-ALL DP cells, include numerous genes that are known to be critical for NOTCH (e.g., *Dtx1*, *Notch1*, *Hes1*, *Hey1* and *Nrarp*), pre-TCR (e.g., *Ptcra*), cytokine and apoptosis pathways (Figure 6C, Table S3). Down-regulation of some direct targets of the ICN1 oncogene (*Dtx1*, *Hes1* and *Hey1*) as a result of *mir-181ab1* deletion was confirmed by quantitative PCR analyses (Figure S3K). Expression of genes in the other clusters (II–IV) was less impacted by *mir-181ab1* deletion, and these clusters are not enriched for known NOTCH pathway genes. Gene set enrichment analyses also confirmed that ICN1-controlled gene sets were effectively reversed to basal or near basal levels in the absence of *mir-181ab1* (Figure 6D). Overall, loss of *mir-181ab1* had drastic effects on the expression of the ICN1-controlled gene set and reverted a significant portion of them back to the levels of normal DP cells (Figure 6A, B). However, Sylamer analyses did not reveal significant enrichment of 7-mer or 8-mer miR-181a seed sequences among the up-regulated genes (Figure 6E). Given that over 25% of ICN1-regulated genes (∼550, >2-fold, *p*<0.01, Table S3) were predicted to be targets of miR-181a by TargetScan, PicTar or miRanda (Figure 6A, indicated by arrow), it is likely that *mir-181ab1* deletion affects the expression of many downstream NOTCH targets. Together, these results suggest that *mir-181ab1* plays a critical role in potentiating NOTCH oncogenic signals.

### *mir-181ab1* dampens negative feedback regulators of NOTCH and pre-TCR pathways

*mir-181ab1* deletion did not completely block NOTCH oncogenic signaling as it did not completely revert the expression of many cluster I genes to the level observed in normal DP cells (Figure 6C). This observation suggests that *mir-181ab1* deletion may have dampened NOTCH oncogenic signals by permitting higher expression of negative feedback molecules, which results in a reduction in NOTCH signaling as indicated by lower induction of NOTCH target genes. Thus, *mir-181ab1* may mediate its effects on the ICN1 oncogenic program through dampening the negative feedback loops controlled by *Notch1* oncogenes. We found that many known negative regulators of the NOTCH signaling pathway, such as *Nrarp*, *Numb*, *Numb-like*, *Hes6* and *Lunatic Fringe* (*Lfng*) mRNAs, contain multiple putative miR-181a binding sites in their 3′ UTR regions (Figure S4A). We devised a biological screen to identify the functionally relevant targets using the OP9-DL1 co-culture assay (Figure S2C). If candidate targets are functionally relevant, ectopic expression of the miR-181a-insensitive version of the targets (binding sites absent or mutated) in thymic progenitor cells should inhibit or dampen the effects of miR-181a on normal DP cell development, resulting in a phenotype that opposes that of ectopic expression miR-181a. In contrast, if candidate targets are functionally irrelevant, ectopic expression of the miR-181a-insensitive version of the targets should have no such effects.

**Figure 7:**
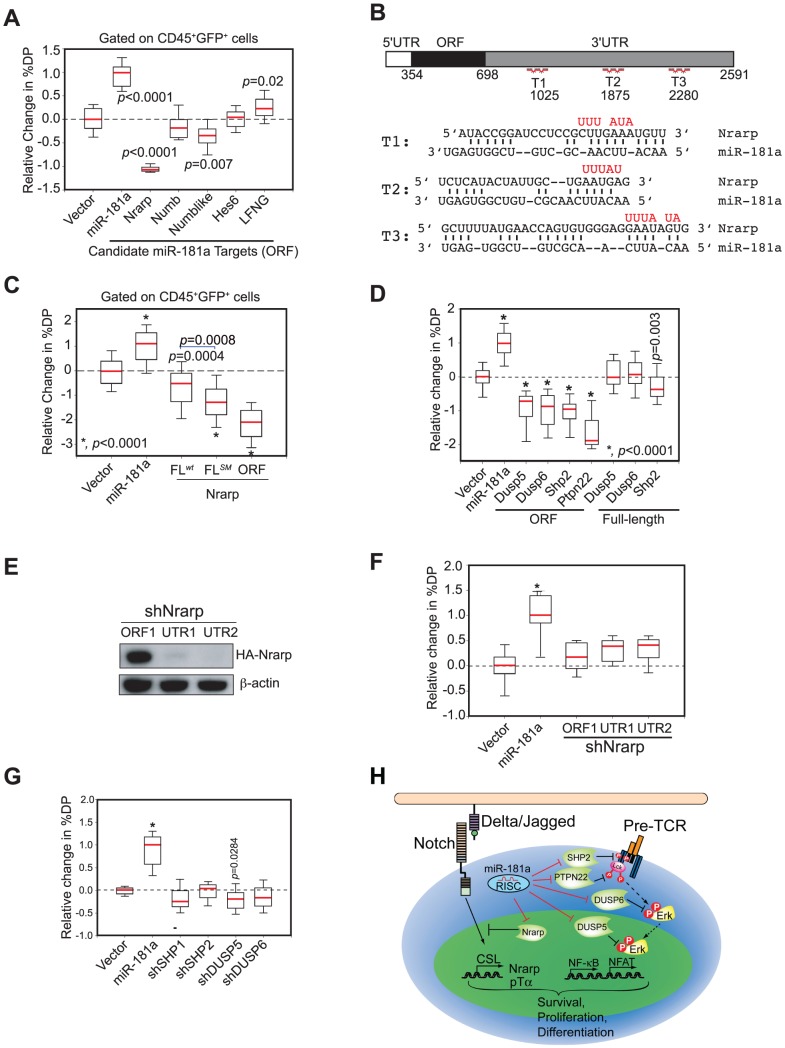
*mir-181ab1* controls the negative feedbacks in NOTCH1 and pre-TCR pathways during normal thymocyte development. (A) The coding regions of individual candidate targets, which lack all or a majority of predicted miR-181a binding sites, were expressed in thymic progenitors, and their effects on DP cell development were determined by using the OP9-DL1 assay (box plots, n = 12, representative analysis of three independent experiments shown). (B) Wild-type and mutant (red) miR-181a target sites in the *Nrarp* 3′UTR (T1, T2, T3) and the predicted base pairings between mouse *Nrarp* and miR-181a. (C) Functional regulation of *Nrarp* by miR-181a during early T cell development. The *Nrarp* gene with a wild-type full-length UTR (FL*^wt^*) or a full-length UTR with the miR-181a-binding site mutated (FL*^mut^*) were ectopically expressed in thymic progenitors and tested for effects on early thymocyte development (box plots, n = 48; replicates pooled from four independent experiments). (D) miR-181a-sensitive (ORF+3′UTR) and miR-181a-insensitive (ORF only) phosphatases were ectopically expressed in thymic progenitors and cultured over OP9-DL1 stromal cells to examine their effects on DP development (*, *p*<0.0001). (E) *Nrarp* shRNAs suppress expression of Nrarp protein as determined by western blot analyses. (F) Effects of *Nrarp* shRNAs on DP cell development in the OP9-DL1 assay (*, *p*<0.05). (G) Effects of shRNAs targeting *SHP1*, *SHP2*, *DUSP5* or *DUSP6* on DP cell development in the OP9-DL1 assay. Data in (F) and (G) are displayed as a relative change in % DP in box plots (n = 12, representative of three independent experiments, *, *p*<0.0001). (H) Schematic diagram of the mechanism by which miR-181a may regulate normal T cell development.

We found that ectopic expression of only the open reading frame (ORF) of *Nrarp* caused strong inhibition of DP thymocyte development; ectopic expression of the *Numb-like* ORF had only a slight effect (∼25% reduction); and ectopic expression of *Numb-like*, *Numb*, *Hes6* or *Lfng* ORFs had no significant effects on DP cell development (Figure 7A and see Figure S4B for representative FACS plots). Thus, consistent with the observed function of Nrarp during early thymocyte development [26], these results demonstrate that *Nrarp* mRNA is likely a functional target of miR-181a in early thymocyte development. Further epistatic analyses showed that expression of *Nrarp*-FL*^wt^*, which contains the full-length *Nrarp* 3′UTR and intact miR-181a binding sites, had limited suppressive activity (Figure 7B, C, and see Figure S4C for representative FACS plots). However, the suppressive activity of *Nrarp*-FL on T cell development significantly increased when the predicted pairings to the miR-181a seeds were abrogated. Moreover, the *Nrarp* ORF had much stronger suppressive activity than did *Nrarp*-FL*^SM^*, suggesting that there may be cryptic miR-181a binding sites in the 3′ UTR of *Nrarp*. All three *Nrarp* expression constructs produced similar levels of *Nrarp* transcripts in a miR-181a-negative cell line as demonstrated by qPCR analyses (Figure S4D). Thus, the differential functional activities observed are not due to inherent differences in *Nrarp* mRNA levels. Since endogenous *Nrarp* mRNAs are present in these assays (Figure S4E), these results demonstrate that *Nrarp* transcripts are suppressed by endogenous miR-181a in early thymocytes. Together, our data show that the predicted miR-181a binding sites in the *Nrarp* 3′ UTR (Figure 7B) are targeted by endogenous miR-181a during early T cell development.

We previously showed that miR-181a potentiates TCR signaling by suppressing the expression of multiple phosphatases, including *Dusp5*, *Dusp6*, *Shp2* and *Ptpn22*, in DP and mature T cells [16]. Since pre-TCR and TCR pathways share common signaling molecules and, more importantly, NOTCH and pre-TCR signaling act synergistically to promote early T cell and T-ALL development [23], [27], miR-181a may also regulate pre-TCR signaling during these developmental processes. Indeed, we found that ectopic expression of the coding regions (miR-181a-insensitive forms) of *Dusp5*, *Dusp6*, *Shp2* and *Ptpn22* efficiently inhibited the development of DP thymocytes, whereas expression of the full-length cDNA versions (miR-181a-sensitive forms) of these phosphatases had little or no effect (Figure 7D and see Figure S4F for representative FACS plots). Since the mRNA transcripts of these phosphatases are readily detectable in various thymic progenitor populations (Figure S4E), and they are validated miR-181a targets in DP and mature T cells [16], these results demonstrate that these phosphatase transcripts are also suppressed by the endogenous miR-181a during early T cell development. Importantly, expression of shRNAs targeting *Nrarp*, *Shp2*, *Dusp5* or *Dusp6* genes did not recapitulate the phenotype of miR-181a ectopic expression in early T cell development (Figure 7E–G) despite the fact that these shRNAs can suppress the expression of corresponding proteins more effectively than miR-181a [16]. Together, these results demonstrate that the effects of miR-181a on early T cell development are mediated through the regulation of multiple negative feedback regulators in both NOTCH and pre-TCR signaling pathways (Figure 7H).

### miR-181a contributes to the maintenance of NOTCH oncogenic activity in T-ALL cells

**Figure 8:**
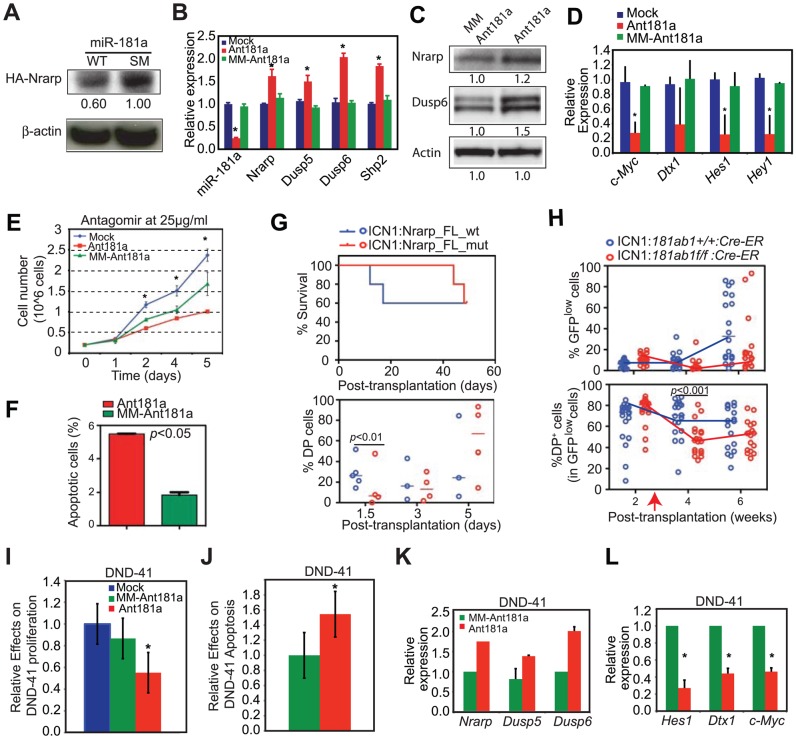
Targets of miR-181a in T-ALL cells and the effects of miR-181a inhibition in T-ALL cells. (A) Western blot analyses were used to determine the levels of HA-tagged Nrarp protein in T6E cell lines expressing wild-type miR-181a or miR-181a*^SM^* (normalized to *β*-actin loading control). (B–D) Levels of miR-181a target expression determined by (B) qPCR or (C) western blot analyses and (D) *Notch1* target expression in mock-treated T6E cells or cells treated with 10 µg/ml antagomir against miR-181a (Ant181a) or mismatch control (MM-Ant181a) for 48 hours (mean ± SD, n = 3, *p<0.05). (E and F) Effects of Ant181a and MM-Ant181a on T6E leukemia cell (E) proliferation and (F) apoptosis (mean ± SD, n = 3, *, *p*<0.05). (G) Kaplan-Meier survival curves and the percentages of ICN1-infected DP cells show the effects of Nrarp target expression on ICN1-induced T-ALL development (5 mice/group). Sorted ICN1-BM cells from primary recipient mice were infected with the same titer of virus expressing either *Nrarp*-FL*^mut^* (miR-181a-insensitive) or *Nrarp*-FL*^w^* ^t^ (miR-181a-sensitive) and transplanted into lethally irradiated recipients (5 mice/group). (H) Effects of conditional deletion of *mir-181ab1* on the T-ALL development at 2 weeks after leukemia induction (see also Figures S8A, B). Percentages of GFP^low^ and GFP^low^ DP cells in the peripheral blood of recipient mice transplanted with either ICN1:*181ab1^+/+^/Cre-ER* BM cells or ICN1:*181a1b1^f/f^/Cre-ER* BM cells were determined by FACS analyses (20 mice/group). Red arrow indicates the initiation of CreER-mediated *mir-181ab1* deletion. (I and J) Effects of Ant181a and MM-Ant181a (25 µg/ml) on human T-ALL DND41 cell (I) proliferation and (J) apoptosis after 96 h in culture (mean ± SD, n = 4, *, *p*<0.05). (K and L) Levels of (K) miR-181a targets and (L) *Notch1* target expression in DND41 cells treated with Ant181a (25 µg/ml) for 48 hours (mean ± SD, n = 3, *p<0.05).

Since both NOTCH and pre-TCR pathways are important for T-ALL development [22], [23] and can each be targeted for T-ALL treatment [28], [29], the above findings (Figure 7A–D) suggest that *mir-181ab1* controls similar pathways in T-ALL cells and in normal DP cells. We went on to examine whether miR-181a contributes to the maintenance of NOTCH oncogenic activity in T-ALL cells by altering expression of similar targets in mouse and human T-ALL cells. First, we stably expressed wild-type miR-181a (miR-181a^WT^) in T6E cells, a murine T-ALL cell line [30]. As shown by western blot analyses, expression of miR-181a^WT^ resulted in ∼40% less HA-tagged Nrarp from a full-length *Nrarp* cDNA than observed in cells that expressed a seed mutant miR-181a (Figure 8A). Second, transient inhibition of miR-181a expression in T6E cells with antagomirs resulted in up-regulation of *Nrarp* (by 61%), *Dusp5* (by 50%), *Dusp6* (by 100%) and *Shp2* (by 84%) mRNAs compared to cells treated with the mismatched control (Figure 8B). Thus, miR-181a dampens expression of at least some of the same targets in T6E cells as it does during normal thymocyte development (Figure 7). Finally, in the T6E cells treated with antagomir-181a, we observed increased Nrarp and DUSP6 protein expression (Figure 8C), down-regulation of expression from *Notch1* controlled targets including *c-Myc*, *Dtx1*, *Hes1* and *Hey1* (Figure 8D), a decrease in proliferation (Figure 8E), and an increase apoptosis (Figure 8F). Further supporting the observations made in T6E cells, we noted that expression of *Nrarp*-FL*^mut^* (an miR-181a-insensitive mRNA) but not *Nrarp*-FL*^w^* ^t^ (miR-181a-sensitive) in T-ALL DP cells from primary recipients suppressed the development of T-ALL DP cells and tumorigenic potential in secondary recipients (Figure 8G). Moreover, induced deletion of *mir-181ab1* caused a significant and persistent decrease in the DP leukemia cell population with low levels of ICN1 expression (GFP^low^ DP cells) in T-ALL mice (Figure 8H). Finally, we examined whether miR-181a contributes to the maintenance of NOTCH oncogenic activity in human T-ALL cells. We found that antagomir inhibition of miR-181a in the human T-ALL cell line DND41 [31] had effects similar to those we observed in murine T6E T-ALL cells (Figure 6C, 8B–D and S3K). Antagomir miR-181a treatment of DND-41 cells caused a reduction in cell proliferation (Figure 8I), an increase of apoptotic cells in the culture (Figure 8J), up-regulation of miR-181a targets (Figure 8K), and down-regulation of Notch targets (Figure 8L). Together, these findings demonstrate that miR-181a contributes to the maintenance of human and mouse T-ALL cells by dampening the negative feedbacks and potentiating NOTCH and pre-TCR signals (Figure S5), suggesting that miR-181a may be a therapeutic target in human T-ALL.

## Discussion

In this study, we examined the roles of three *mir-181* genes, *mir-181ab1*, *mir-181ab2* and *mir-181cd*, in normal thymocyte development and in T-ALL development. We found that deletion of *mir-181ab1*, but not *mir-181ab2* and *mir-181cd*, effectively inhibited NOTCH1-induced T-ALL. Moreover, the effects of *mir-181ab1* deletion on *Notch1* oncogenic potential depend on the expression levels and the signaling strength of the oncogenes. In particular, we showed that *mir-181ab1* deletion inhibits the oncogenic activity of P12ΔP — one of the strongest NOTCH1 mutants identified in human T-ALL patients [25] — indicating that targeting *mir-181ab1* may effectively inhibit the tumorigenic potential of other human *Notch1* mutants. Our results demonstrate that *mir-181ab1* can regulate the strength and threshold of *Notch1* oncogenic activity. It is important to note that deletion of *mir-181ab1* had a quantitative effect on normal development that was sufficient to dampen *Notch1* oncogenic potential and dramatically improve mortality in T-ALL mice (Figure 4). Together with our previous study showing that miR-181a functions as a rheostat in regulating the strength and threshold of TCR signaling and T cell sensitivity to antigen [16], these results illustrate a general model of miRNA function in controlling the strength and threshold of receptor signaling by regulating multiple targets during normal and oncogenic developmental processes. These findings support the notion that quantitative regulation of oncogenic signal strength can be sufficient to block cancer development [8] and demonstrate that miRNAs may be effective therapeutic targets [9].

Our comparative analyses revealed that the pathways controlled by *mir-181ab1* are not of equal importance in normal thymic progenitor cells and T-ALL DP cells (Figure 2 – 4). The fact that co-expression of *mir-181a-1* together with ICN1 did not significantly potentiate the oncogenic activity of ICN1 (Figure S6) implies that endogenous *mir-181ab1* and the pathways it controls may be sufficient to potentiate *Notch1* oncogene signaling. Clearly the effects of *mir-181ab1* deletion were compensated for during normal thymic progenitor development but not during T-ALL development (Figure 2 – 4). It is possible that normal vertebrate immune systems, which have central roles in controlling host immunity and homeostasis, may have many intrinsic mechanisms to withstand many forms of perturbations and can compensate for the *mir-181ab1* deletion. In contrast, T-ALL cells may be more reliant on the pathways controlled by *mir-181ab1* and homeostatic mechanisms most likely do not exist in T-ALL cells. Our findings illustrate that comparative studies on the pathways utilized by normal cells and tumor cells can reveal how tumorigenic pathways may be selectively inhibited with limited damage to normal tissues. Since germline *mir-181ab1* knockout mice are viable and have no noticeable defects for up to twelve months, inhibition of *mir-181ab1* activity should block NOTCH1-induced tumorigenesis without significant side effects. However, since miR-181 miRNAs are highly expressed in brain and muscle tissue [15], further studies should be carried out to examine the function of *mir-181* alleles in the development and function of non-hematopoietic tissues and organs.

The ability to regulate multiple targets enables a miRNA to mediate its biological function by controlling varied gene sets in different cell types. This results in the expansion of regulatory complexities conferred by the same set of protein-coding genes during normal lineage development and tumorigenesis. In this study, dissecting the multi-target networks controlled by miRNAs during normal thymic T cell development and NOTCH-induced T-ALL allowed us to unravel the downstream molecular networks that contribute to normal thymic T cell development and NOTCH-induced tumorigenesis. Early studies have elegantly shown that NOTCH and pre-TCR signals play critical roles during normal thymic progenitor cell development and T-ALL development [22], [23]. However, neither NOTCH nor pre-TCR signals can be targeted effectively for treatment of T-ALL with inhibitors like GSI or cyclosporine, respectively, due to weak therapeutic effects and severe toxicity [28], [29]. Our finding that *mir-181ab1* modulates both normal and leukemogenic DP cell development in part by repressing the negative feedback regulators of NOTCH and pre-TCR signaling pathways (Figure 2 – 4, 6, 7) demonstrates that it is possible to target *mir-181ab1* to inhibit both NOTCH and pre-TCR signals simultaneously and effectively block T-ALL development. Given the extensive list of predicted *mir-181ab1* targets (Figure 6A and Table S3), we could not exhaustively identify all functional targets for *mir-181ab1* in normal and T-ALL DP cells and therefore, loss of *mir-181ab1* may also compromise other pathways that are required for NOTCH-induced T-ALL. A recent study by Cichocki *et al.* suggested that miR-181 might influence human natural killer cell development by targeting NLK, a negative regulator of WNT and NOTCH signaling [32]. We found that NLK mRNA is expressed in DP T-ALL cells. However, ICN1 expression did not change the *NLK* mRNA levels in DP cells and loss of *mir-181ab1* did not alter *NLK* mRNA levels in DP cells (Table S2). Although these observations suggest that NLK might not be regulated by miR-181a in T-ALL cells at the mRNA level, it will be interesting to explore whether NLK is regulated by miR-181a at a translational level. Of note, according to various target prediction program [33], [34] and functional analyses, miR-181a may regulate similar targets in human T-ALL cells (Figure 8), including *Nrarp* (Figure S7) and various phosphatase mRNAs. Many validated miR-181a targets in mouse cells Together, these findings illustrate that study of miRNA function in cancer will help to elucidate the molecular networks required for oncogenic transformation and shed insights into the downstream networks that can be targeted to inhibit tumor development.

Interestingly, our results showed that the miR-181 family miRNAs are not functionally equivalent during normal and T-ALL development even though all have the same seed nucleotides. Deletion of *mir-181ab2* and *mir-181cd* has different effects on T-ALL development, and neither allele could compensate for the loss of *mir-181ab1* (Figure 5, and Figure S3F, G). There are several likely causes of the differential effects of loss of *mir-181ab1*, *mir-181ab2* and *mir-181cd* on normal DP cell development and ICN1-induced T-ALL development. First, differences in expression levels in DP leukemia cells may contribute to their various effects on T-ALL development (Figure 5A, 5B). Second, their differential expression patterns in various thymic T cell populations may underlie their varied effects on normal thymocyte and T-ALL development [14]–[18]. Lastly, the extended nucleotide differences between miR-181a, miR-181b, miR-181c, and miR-181d and their coding genes may contribute to their varied effects on T-ALL development [21]. Further in-depth analyses will be needed to examine these possibilities and to elucidate the mechanisms through which various *mir-181* alleles mediate differential activities during normal thymocytes and T-ALL development. Of interest, mice with all three *mir-181* alleles deleted could not be generated from crossing the single knockouts presumably due to early lethality (data not shown), suggesting that there might be toxicity if all three *mir-181* alleles are targeted simultaneously for T-ALL treatment. Thus, the fact that *mir-181ab1*, but not *mir-181ab2* and *mir-181cd*, controls the development of normal DP cells and T-ALL DP cells suggests that specific targeting *mir-181ab1* may be an effective approach to inhibition of NOTCH-induced T-ALL development.

## Methods

See Text S1 for more detailed descriptions.

### Mice

C57BL/6J or 129/SvJ mice were obtained from Jackson Laboratory and maintained at the Stanford University Department of Comparative Medicine Animal Facility in accordance with National Institutes of Health guidelines. Knockout strains are maintained on either a 129 background or mixed B6.

### Bone marrow transplantation

Hematopoietic stem/progenitor BM cells were isolated from mice treated with 5-fluorouracil (5′ FU) and infected with MigR1, ICN1, P12ΔP or ICN1:*mir-181a-1* retroviruses. Secondary transplantation was carried out by sorting GFP^+^DP^+^ BM cells from primary T-ALL recipient mice. A mixture of 1×10^5^ infected cells and 1×10^5^ total BM cells (supporting cells) were intravenously injected into lethally irradiated (9.5 Gy) 129-strain recipient mice (∼6 weeks old). To assess T-ALL development, peripheral blood samples were acquired from the recipient mice at various time points after transplantation and analyzed by FACS to determine the percentage of DP cells. The Kaplan-Meier estimator was used to determine the median rate of survival. The *p* values were determined using the Mantel-Cox test.

### OP9-DL1 assay for *in vitro* T cell differentiation

Sorted or total thymocytes were cultured and differentiated on OP9-DL1 cells as described [20], [21]. FACS analyses were carried out to determine the effects of miRNAs on DP thymocyte development. Anti-CD45 antibody staining and/or FSC/SSC gating were used to differentiate infected thymocytes and GFP^+^ stromal cells. The results are summarized in box-plots to describe the % DP cells from more than 12 replicate cultures. The ends of the boxes define the 25^th^ and 75^th^ percentiles; a line indicates the median and bars define the 5^th^ and 95^th^ percentiles. In some cases, the results were normalized so that the negative control had a median activity of 0 and the wild-type *mir-181a-1* expressing vector had a median activity of 1. Due to the heterogeneous nature of the thymic progenitor cells and intrinsic variation between the batches of mice used, normalization allowed for comparison among the independent repeats. Mann-Whitney rank sum tests were performed to determine statistical significance.

### Microarray expression profiling and miRNA target analyses

Total RNAs were labeled using Illumina's Total Prep RNA Amplification Kit and hybridized to Illumina MouseRef-8_V2 BeadChips according to the manufacturer's instructions. Data were normalized using the quantile method (the Bioconductor *lumi* package). SAM analyses were performed to select differentially expressed genes (>2-fold, *p*<0.01). Gene expression patterns were determined by hierarchical clustering with Pearson correlation as similarity metric. Heatmaps were generated with the Gplots R package. Ingenuity Pathways Analyses were carried out to determine the functional pathways within gene clusters. Gene set enrichment analyses (GSEA) were used to determine the effects of *mir-181ab1* deletion on the gene sets up- or down-regulated by ICN1 in T-ALL DP cells (permutation = 1000). miR-181a targets with perfect “seed” matches were identified using TargetScans 5.1 (http://www.targetscan.org/), PicTar (http://pictar.mdc-berlin.de/) or miRanda (http://www.microrna.org/microrna/home.do). Alternatively, the m-fold program was used to identify putative miR-181a binding sites on selected target mRNAs. Sylamer analyses were carried out to determine the enrichment of seed matches among the genes up- or down-regulated in the absence of *mir-181ab1* [35]. Control seeds were from other miRNAs in the miRBase (release 12).

## Supporting Information

Figure S1 Generation of conditional *mir-181* alleles by gene targeting in ES cells. (A–C) Schematic representation of the target strategies for generating conditional (A) *mir-181a-1/b-1 (mir-181ab1)*, (B) *mir-181a-2/b-2 (mir-181ab2)* and (C) *mir-181c/d (mir-181cd)* alleles. Restriction enzyme sites and probes for Southern blot analyses are also indicated. DT: diphtheria toxin cassette. (D–F) Southern blot analyses of mouse embryonic stem cell clones with targeted (D) *mir-181ab1*, (E) *mir-181ab2* and (F) *mir-181c/d* alleles. Genomic DNA from targeted ES clones was digested with BglII, EcoRV, and XbaI and probed with corresponding probes. (G) Schematics depicting the *mir-181* loci and known protein-coding genes within the regions.
(PDF)

Figure S2 Effects of loss of mir-181ab1 on normal thymocyte development. (A and B) Representative FACS plots showing the effects of *mir-181ab1* germline deletion on (A) DN T cell subsets and (B) ETPs. (C) Schematic of the modified OP9-DL1 co-culture assay. (D) Effects of seed mutations on miR-181a function in early thymocyte development (box plots, n = 12, representative of four experiments). Representative FACS plots are shown here. (E) Northern blot analyses of mature miRNA expression from the wild-type and seed mutant (SM) miR-181a expression constructs. (F and G) The effects of *mir-181ab1* deletion on cellularity (F) and apoptosis (G) of the OP9-DL1 culture. (H) Deletion of the floxed *mir-181a-1/b-1* alleles by Cre/GFP virus expression determined by PCR analyses. (I) miR-181a expression in DP thymocytes infected with Cre/GFP viruses determined by miRNA qPCR analyses. (J) Down-regulation of miR-181a expression during DN3a to DN3b transition determined by miRNA qPCR analyses. (K) Intracellular TCR-β expression in DN3 populations and DN4 thymic progenitor populations before and after *mir-181ab1* deletion.
(PDF)

Figure S3 Effects of loss of *mir-181ab1* on ICN1-induced T-ALL development. (A) Percentage of ICN1 infected cells (GFP^+^) and percentage of DP leukemia cells among the ICN1-infected cells in the bone marrow of T-ALL mice at various time points after reconstitution determined by FACS analyses. Each line represents the changes in percent of GFP^+^ and GFP^+^DP cells of individual recipient. (B–D) Percentage of GFP^+^ and GFP^+^DP cells in (B) lymph nodes, (C) lungs and (D) liver of recipient mice at 2, 4 and 6 weeks after reconstitution with ICN1-infected hematopoietic stem/progenitor cells from mice with either wild-type or *mir-181ab1* null alleles (* *p*<0.05, **p<0.01, ***p<0.001). (E) Loss of *mir-181ab1* does not compromise the long-term reconstitution potential of hematopoietic stem/progenitor cells. FACS analyses of GFP^+^ cells in the peripheral blood of mice transplanted with *mir-181ab1^+/+^* and *mir-181a1b1^−/−^* bone marrow progenitor cells transduced with the control vector MigR1. (F and G) Effects of loss of (F) *mir-181ab2* and (G) *mir-181cd* alleles on the median survival of ICN1-induced T-ALL mice. Kaplan-Meier survival curves show the percentage of mice surviving at different time points after T-ALL induction (*p*>0.05). (H) Histological analysis (H&E staining) of T-ALL mice at 2 and 4 weeks after transplantation. Representative sections from BM, spleen and thymus of ICN1-induced T-ALL mice are shown. Bars equal 20 microns (bone marrow), 100 microns (spleen) and 200 microns (thymus). (I) FACS analyses were carried out to determine the changes in GFP expression levels in P12ΔP-infected PB cells from 3 to 21 weeks after transplantation for those two P12ΔP:*181ab1^−/−^* T-ALL mice that died of T-ALL. (J) Comparison of ICN1 expression levels between ICN1:*181ab1^+/+^* and ICN1:*181ab1^−/−^* BM cells in both GFP^high^ and GFP^low^ cells population at 4weeks after transplantation. (K) Effects of loss of *mir-181ab1* on expression of Notch target genes (*Hes1*, *Deltex1*, *Hey1*) in DP leukemia cells as determined by qPCR analyses (mean ± SD, n = 3, *p<0.05).
(PDF)

Figure S4 *mir-181a1b1* targets in normal thymic progenitors and T-ALL cells. (A) Schematic diagram of predicted miR-181a binding sites in mRNAs of some of the negative regulators of Notch signaling. *Numb*, *Numb-like*, *Hes6*, and *LFNG* are presented schematically to include the 5′UTR (yellow), ORF (orange), and 3′UTR (blue). The approximate locations of the predicted miR-181a pairing site is presented as a bold red line and numbered. Nucleotide numbers that define the three regions correspond to their respective GenBank accession number. (B) The coding regions of individual candidate targets, which lack all or a majority of predicted miR-181a binding sites, were expressed in thymic progenitors, and their effects on DP cell development were determined by using the OP9-DL1 assay. Representative FACS plots for Figure 7A are shown here. (C) Wild-type full-length (FL*^wt^*) and the mutant full-length (FL*^mut^*) Nrarp were expressed in thymic progenitors to determine the effects of these predicted miR-181a binding sites on early thymocyte development. Representative FACS plots for Figure 7C are shown here. (D) Constructs harboring the wild-type full-length (FL*^wt^*), the mutant full-length (FL*^mut^*), or the coding region (ORF) of *Nrarp* produce similar levels of *Nrarp* transcripts (determined by quantitative RT-PCR, mean ± SD, n = 3). (E) The relative levels of miR-181a and its cognate targets in DN1–4 and DP thymocytes were determined by miRNA or mRNA qPCR analyses and normalized to the corresponding levels in DP cells (mean ± SD, n = 3). (F) miR-181a sensitive (ORF+3′UTR) and insensitive (ORF only) phosphatases were ectopically expressed in thymic progenitors and cultured over OP9δ stromal cells to examine their effects on DP development. Representative FACS plots for Figure 7D are shown.
(PDF)

Figure S5 Schematic diagram depicting the proposed model by which *mir-181ab1* contributes to the leukemogenic potential of Notch oncogenes through damping the negative feedbacks in Notch and pre-TCR signaling pathways.
(PDF)

Figure S6 Overexpression of *mir-181a-1* does not potentiate ICN1-induced T-ALL development. (A) The retroviral construct used to co-express ICN1 and *mir-181a-1*. (B) Schematics depicting the experiment. (C) Kaplan-Meier survival curves show the percentages of mice surviving at different time points after reconstituting with BM cells infected with either ICN1/*mir-181a-1* (n = 10 mice) or ICN1/*mir-181a_sm* (n = 10 mice) viruses. (D) Effects of *mir-181a-1* overexpression on the percentage of total ICN1-infected cells (all GFP^+^ cells) and the percent of ICN1-infected DP leukemia cells (GFP^+^DP cells). The *p* values were determined using the Mantel-Cox test (*, *p*<0.05).
(PDF)

Figure S7 Predicted base pairings between human Nrarp and miR-181a.
(PDF)

Figure S8 Inducible deletion of *mir-181ab1* in ICN1-infected DP leukemia cells. (A) Schematics depicting a strategy for inducible deletion of *mir-181ab1* in T-ALL mice. (B) *mir-181ab1* deletion was induced by intraperitoneal injection of tamoxifen (2 mg/kg) every 2 days during a 10-day period. (C) Deletion of *mir-181ab1* in hematopoietic/lymphoid organs was confirmed by PCR analysis at 4, 7 and 12 days after the first tamoxifen injection. At 12 days after the first tamoxifen injection, *mir-181ab1* was deleted from bone marrow, spleen, lymph node and thymus cells.
(PDF)

Table S1 List of hematopoietic and lymphoid populations examined by FACS analyses.
(XLS)

Table S2 Raw data of gene expression profiling of wild-type DP, ICN1-DP, and ICN1_DP_181KO cells. Three independent biological replicates for wild-type DP, ICN1-DP, and ICN1_DP_181KO cells were isolated by FACS sorting from control and ICN1-induced T-ALL recipient mice at 4 weeks after transplantation. Gene expression profiles of the wild-type DP, ICN1-DP, and ICN1_DP_181KO cells were determined by using the Illumina mouse Ref-8_V2 BEADCHIP array.
(XLS)

Table S3 Complete list of ICN1-regulated genes and their differential expression in the absence of mir-181ab1. Hierarchical clustering analyses clustered this set of genes into four groups (I, II, III, IV). Ingenuity Pathways Analyses (Ingenuity Systems, www.ingenuity.com) were carried out to determine whether these genes belong to over-represented canonical pathways (*p*<0.01). miRNA target prediction analyses were also carried out using TargetScan and PicTar and miRanda. The results of pathway and target analyses were denoted as “1” if positive and “0” if negative.
(XLS)

Text S1 Supplemental results and supplemental experimental procedures.
(DOC)
