# Supplementary figures and images for: Modulating the Strength and Threshold of NOTCH Oncogenic Signals by mir-181a-1/b-1
Source: PLoS Genet. 2012 Aug 9;8(8):e1002855. doi: 10.1371/journal.pgen.1002855 (PMC3415433; doi:10.1371/journal.pgen.1002855)

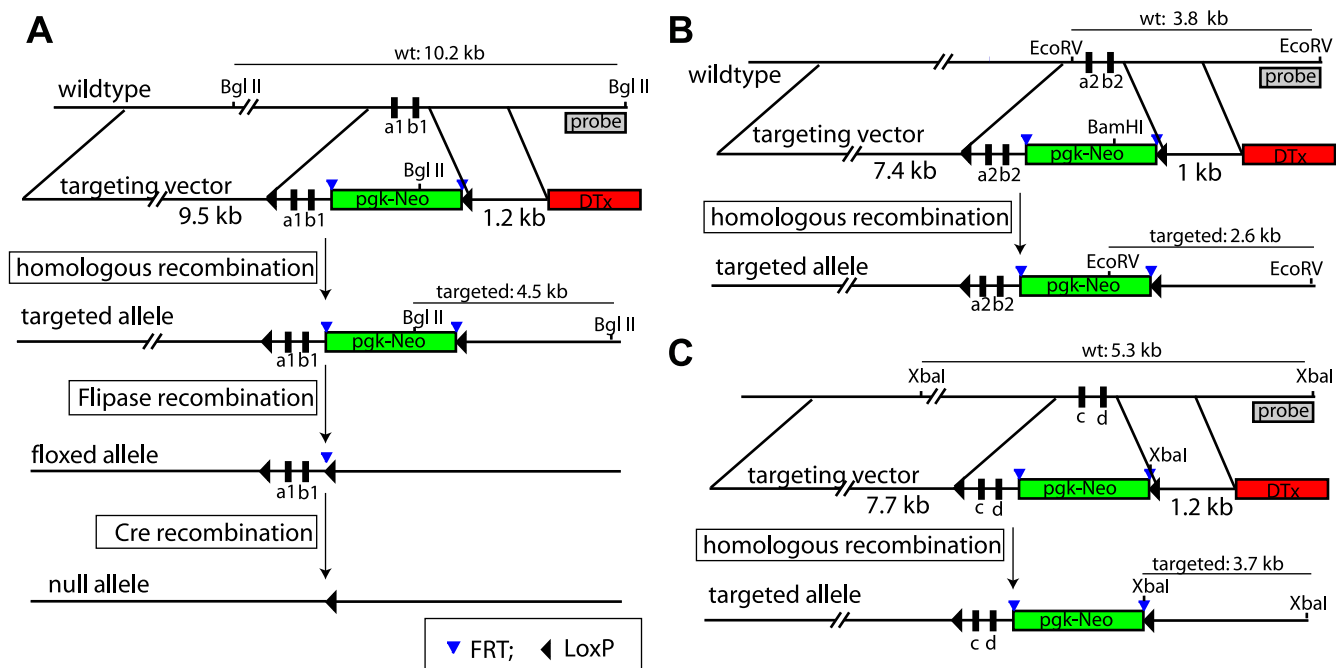

**D** *mir-181ab1* (Bgl II)  
+/+ +/+ +/neo +/neo

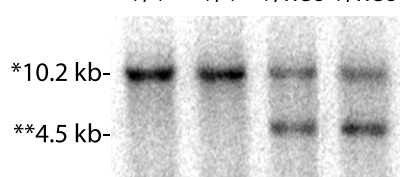

**E** *mir-181ab2* (EcoRV)  
+/+ +/+ +/neo +/neo

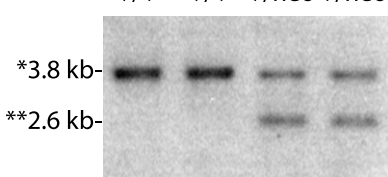

**F** *mir-181cd* (XbaI)  
+/neo +/+ +/neo +/neo

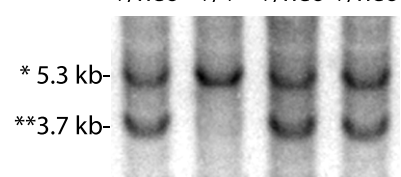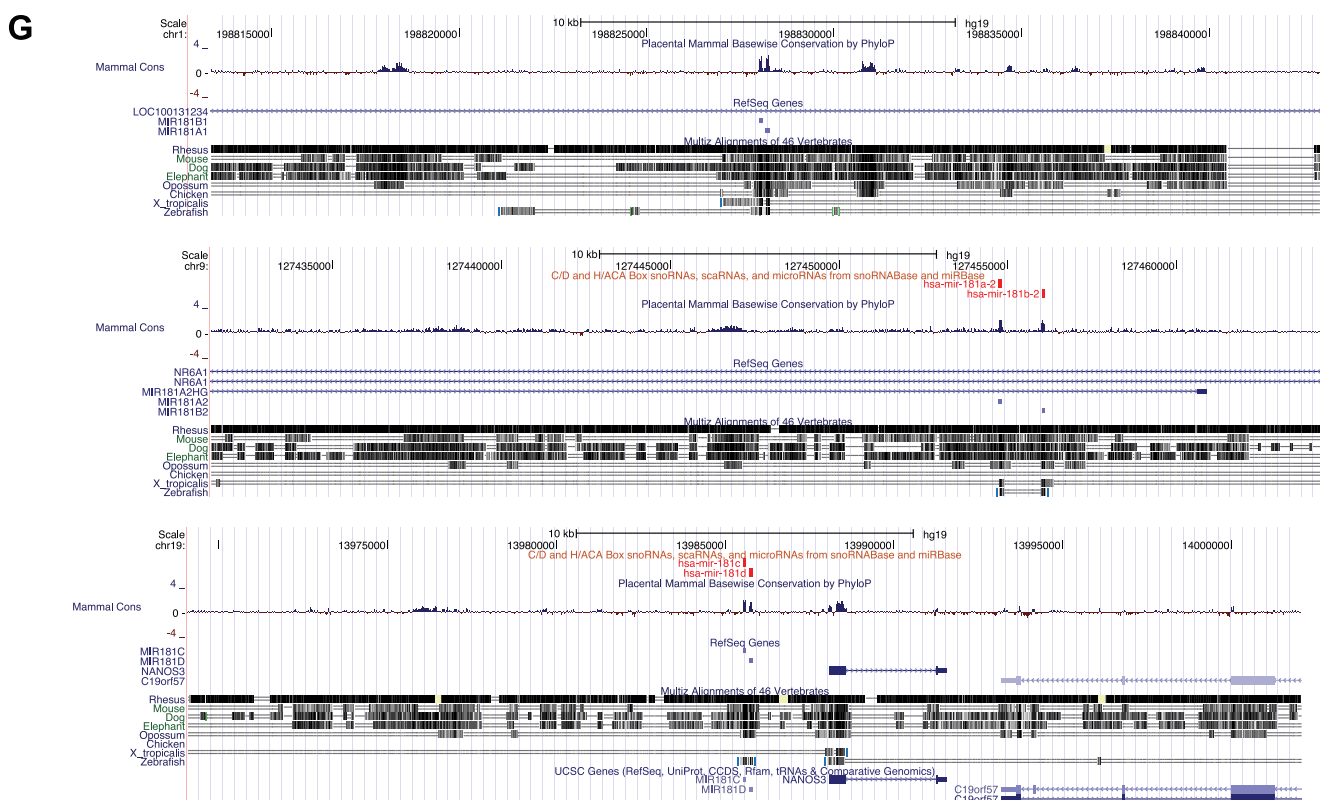

Supplement: Figure S1 — Generation of conditional mir-181 alleles by gene targeting in ES cells. (A–C) Schematic representation of the target strategies for generating conditional (A) mir-181a-1/b-1 (mir-181ab1), (B) mir-181a-2/b-2 (mir-181ab2) and (C) mir-181c/d (mir-181cd) alleles. Restriction enzyme sites and probes for Southern blot analyses are also indicated. DT: diphtheria toxin cassette. (D–F) Southern blot analyses of mouse embryonic stem cell clones with targeted (D) mir-181ab1, (E) mir-181ab2 and (F) mir-181c/d alleles. Genomic DNA from targeted ES clones was digested with BglII, EcoRV, and XbaI and probed with corresponding probes. (G) Schematics depicting the mir-181 loci and known protein-coding genes within the regions. (PDF) [file pgen.1002855.s001.pdf]

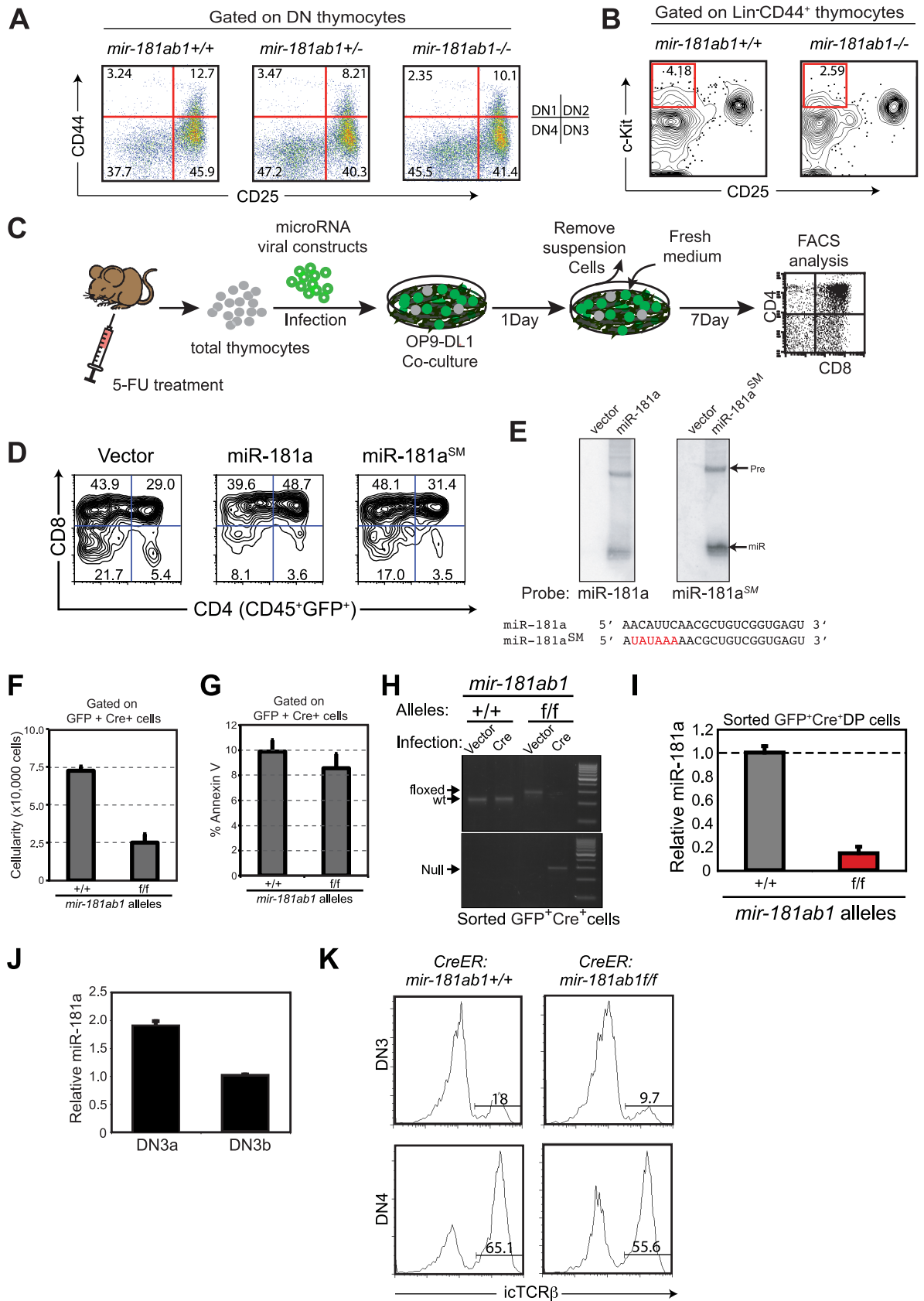

Supplement: Figure S2 — Effects of loss of mir-181ab1 on normal thymocyte development. (A and B) Representative FACS plots showing the effects of mir-181ab1 germline deletion on (A) DN T cell subsets and (B) ETPs. (C) Schematic of the modified OP9-DL1 co-culture assay. (D) Effects of seed mutations on miR-181a function in early thymocyte development (box plots, n = 12, representative of four experiments). Representative FACS plots are shown here. (E) Northern blot analyses of mature miRNA expression from the wild-type and seed mutant (SM) miR-181a expression constructs. (F and G) The effects of mir-181ab1 deletion on cellularity (F) and apoptosis (G) of the OP9-DL1 culture. (H) Deletion of the floxed mir-181a-1/b-1 alleles by Cre/GFP virus expression determined by PCR analyses. (I) miR-181a expression in DP thymocytes infected with Cre/GFP viruses determined by miRNA qPCR analyses. (J) Down-regulation of miR-181a expression during DN3a to DN3b transition determined by miRNA qPCR analyses. (K) Intracellular TCR-β expression in DN3 populations and DN4 thymic progenitor populations before and after mir-181ab1 deletion. (PDF) [file pgen.1002855.s002.pdf]

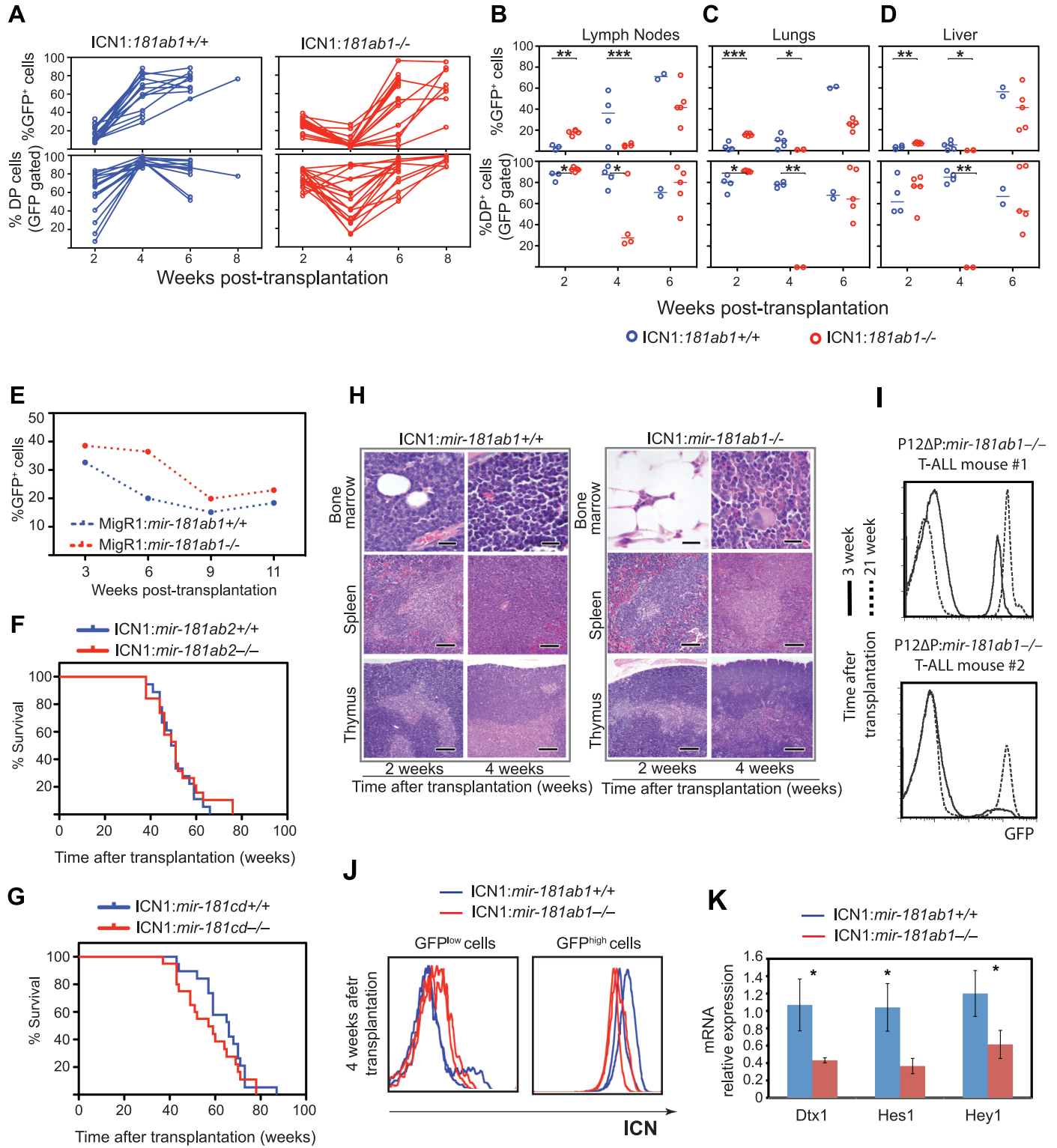

Supplement: Figure S3 — Effects of loss of mir-181ab1 on ICN1-induced T-ALL development. (A) Percentage of ICN1 infected cells (GFP+) and percentage of DP leukemia cells among the ICN1-infected cells in the bone marrow of T-ALL mice at various time points after reconstitution determined by FACS analyses. Each line represents the changes in percent of GFP+ and GFP+DP cells of individual recipient. (B–D) Percentage of GFP+ and GFP+DP cells in (B) lymph nodes, (C) lungs and (D) liver of recipient mice at 2, 4 and 6 weeks after reconstitution with ICN1-infected hematopoietic stem/progenitor cells from mice with either wild-type or mir-181ab1 null alleles (* p<0.05, **p<0.01, ***p<0.001). (E) Loss of mir-181ab1 does not compromise the long-term reconstitution potential of hematopoietic stem/progenitor cells. FACS analyses of GFP+ cells in the peripheral blood of mice transplanted with mir-181ab1+/+ and mir-181a1b1−/− bone marrow progenitor cells transduced with the control vector MigR1. (F and G) Effects of loss of (F) mir-181ab2 and (G) mir-181cd alleles on the median survival of ICN1-induced T-ALL mice. Kaplan-Meier survival curves show the percentage of mice surviving at different time points after T-ALL induction (p>0.05). (H) Histological analysis (H&E staining) of T-ALL mice at 2 and 4 weeks after transplantation. Representative sections from BM, spleen and thymus of ICN1-induced T-ALL mice are shown. Bars equal 20 microns (bone marrow), 100 microns (spleen) and 200 microns (thymus). (I) FACS analyses were carried out to determine the changes in GFP expression levels in P12ΔP-infected PB cells from 3 to 21 weeks after transplantation for those two P12ΔP:181ab1−/− T-ALL mice that died of T-ALL. (J) Comparison of ICN1 expression levels between ICN1:181ab1+/+ and ICN1:181ab1−/− BM cells in both GFPhigh and GFPlow cells population at 4weeks after transplantation. (K) Effects of loss of mir-181ab1 on expression of Notch target genes (Hes1, Deltex1, Hey1) in DP leukemia cells as determined by q [file pgen.1002855.s003.pdf]

**A**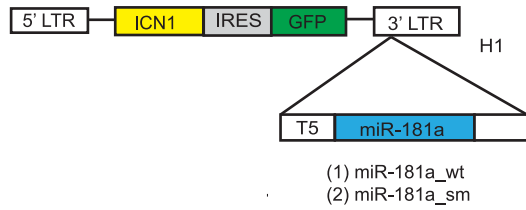**B**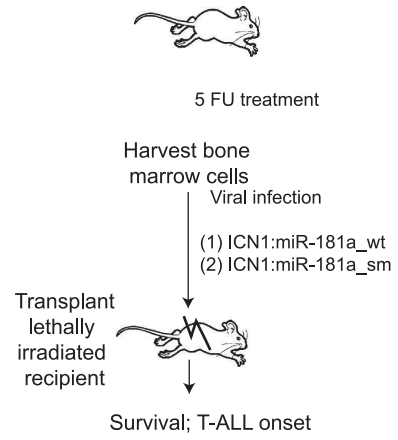**C**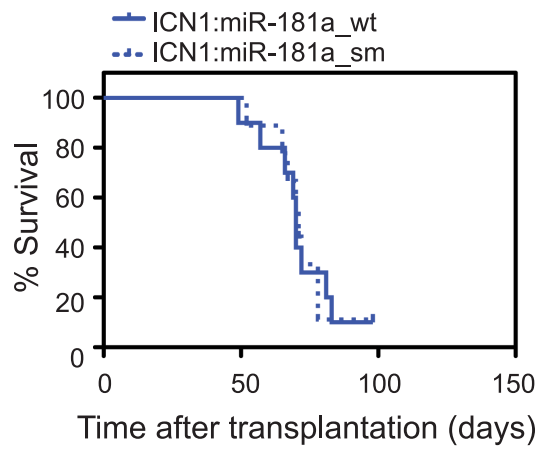**D**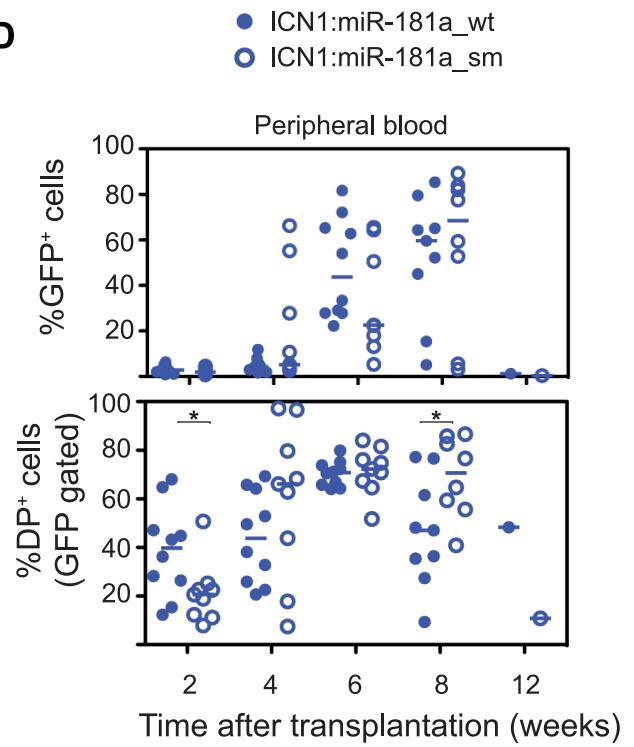

Supplement: Figure S6 — Overexpression of mir-181a-1 does not potentiate ICN1-induced T-ALL development. (A) The retroviral construct used to co-express ICN1 and mir-181a-1. (B) Schematics depicting the experiment. (C) Kaplan-Meier survival curves show the percentages of mice surviving at different time points after reconstituting with BM cells infected with either ICN1/mir-181a-1 (n = 10 mice) or ICN1/mir-181a_sm (n = 10 mice) viruses. (D) Effects of mir-181a-1 overexpression on the percentage of total ICN1-infected cells (all GFP+ cells) and the percent of ICN1-infected DP leukemia cells (GFP+DP cells). The p values were determined using the Mantel-Cox test (*, p<0.05). (PDF) [file pgen.1002855.s006.pdf]

**A**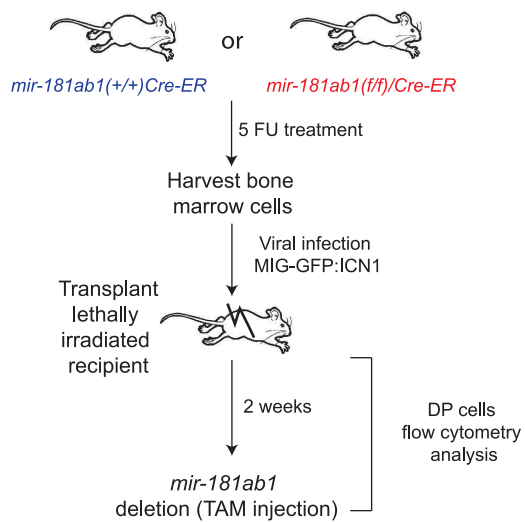**B**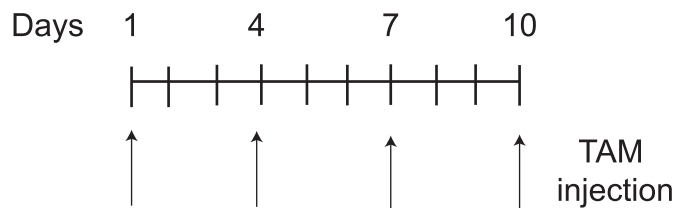**C**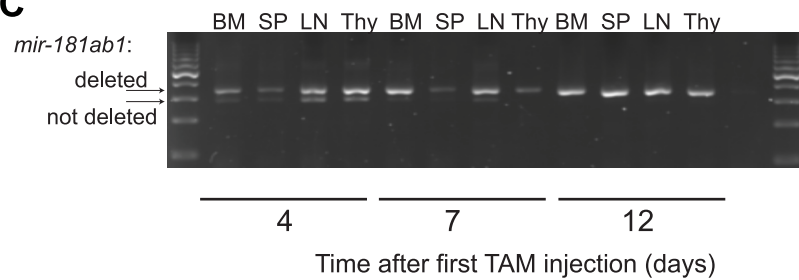

Supplement: Figure S8 — Inducible deletion of mir-181ab1 in ICN1-infected DP leukemia cells. (A) Schematics depicting a strategy for inducible deletion of mir-181ab1 in T-ALL mice. (B) mir-181ab1 deletion was induced by intraperitoneal injection of tamoxifen (2 mg/kg) every 2 days during a 10-day period. (C) Deletion of mir-181ab1 in hematopoietic/lymphoid organs was confirmed by PCR analysis at 4, 7 and 12 days after the first tamoxifen injection. At 12 days after the first tamoxifen injection, mir-181ab1 was deleted from bone marrow, spleen, lymph node and thymus cells. (PDF) [file pgen.1002855.s008.pdf]
