# Supplementary material for: Modulating the Strength and Threshold of NOTCH Oncogenic Signals by mir-181a-1/b-1
Source: PLoS Genet. 2012 Aug 9;8(8):e1002855. doi: 10.1371/journal.pgen.1002855 (PMC3415433; doi:10.1371/journal.pgen.1002855)
Supplement: Figure S4 — mir-181a1b1 targets in normal thymic progenitors and T-ALL cells. (A) Schematic diagram of predicted miR-181a binding sites in mRNAs of some of the negative regulators of Notch signaling. Numb, Numb-like, Hes6, and LFNG are presented schematically to include the 5′UTR (yellow), ORF (orange), and 3′UTR (blue). The approximate locations of the predicted miR-181a pairing site is presented as a bold red line and numbered. Nucleotide numbers that define the three regions correspond to their respective GenBank accession number. (B) The coding regions of individual candidate targets, which lack all or a majority of predicted miR-181a binding sites, were expressed in thymic progenitors, and their effects on DP cell development were determined by using the OP9-DL1 assay. Representative FACS plots for Figure 7A are shown here. (C) Wild-type full-length (FLwt) and the mutant full-length (FLmut) Nrarp were expressed in thymic progenitors to determine the effects of these predicted miR-181a binding sites on early thymocyte development. Representative FACS plots for Figure 7C are shown here. (D) Constructs harboring the wild-type full-length (FLwt), the mutant full-length (FLmut), or the coding region (ORF) of Nrarp produce similar levels of Nrarp transcripts (determined by quantitative RT-PCR, mean ± SD, n = 3). (E) The relative levels of miR-181a and its cognate targets in DN1–4 and DP thymocytes were determined by miRNA or mRNA qPCR analyses and normalized to the corresponding levels in DP cells (mean ± SD, n = 3). (F) miR-181a sensitive (ORF+3′UTR) and insensitive (ORF only) phosphatases were ectopically expressed in thymic progenitors and cultured over OP9δ stromal cells to examine their effects on DP development. Representative FACS plots for Figure 7D are shown. (PDF) [file pgen.1002855.s004.pdf]

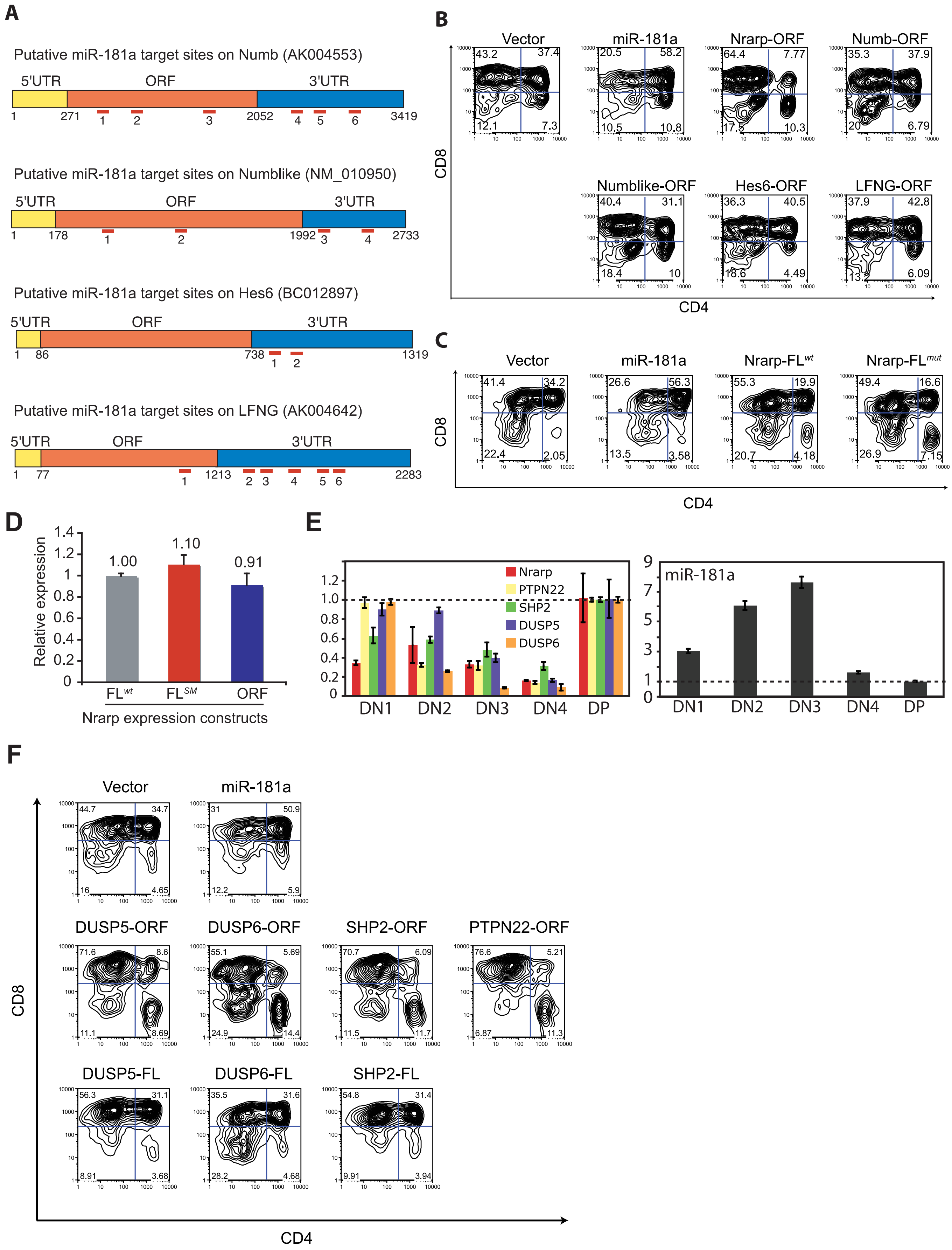
A
Putative miR-181a target sites on Numb (AK004553)
5'UTR ORF 3'UTR
1 271 1 2 3 2052 4 5 6 3419
Putative miR-181a target sites on Numblike (NM_010950)
5'UTR ORF 3'UTR
1 178 1 2 1992 3 4 2733
Putative miR-181a target sites on Hes6 (BC012897)
5'UTR ORF 3'UTR
1 86 738 1 2 1319
Putative miR-181a target sites on LFNG (AK004642)
5'UTR ORF 3'UTR
1 77 1 1213 2 3 4 5 6 2283
B
Vector miR-181a Nrarp-ORF Numb-ORF
Numblike-ORF Hes6-ORF LFNG-ORF
CD8
CD4
C
Vector miR-181a Nrarp-FL^wt Nrarp-FL^mut
CD8
CD4
D
Relative expression
1.00 1.10 0.91
FL^wt FL^SM ORF
Nrarp expression constructs
E
Nrarp
PTPN22
SHP2
DUSP5
DUSP6
DN1 DN2 DN3 DN4 DP
miR-181a
F
Vector miR-181a
DUSP5-ORF DUSP6-ORF SHP2-ORF PTPN22-ORF
DUSP5-FL DUSP6-FL SHP2-FL
CD8
CD4
