# Supplementary material for: Modulating the Strength and Threshold of NOTCH Oncogenic Signals by mir-181a-1/b-1
Source: PLoS Genet. 2012 Aug 9;8(8):e1002855. doi: 10.1371/journal.pgen.1002855 (PMC3415433; doi:10.1371/journal.pgen.1002855)

Dampened NOTCH and Pre-TCR signals without *mir-181ab1*

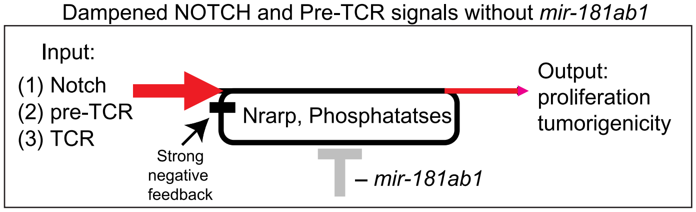

Potentiated NOTCH and Pre-TC signals with *mir-181ab1*

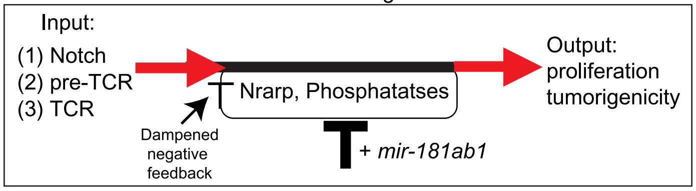

Supplement: Figure S5 — Schematic diagram depicting the proposed model by which mir-181ab1 contributes to the leukemogenic potential of Notch oncogenes through damping the negative feedbacks in Notch and pre-TCR signaling pathways. (PDF) [file pgen.1002855.s005.pdf]
