# Supplementary material for: Modulating the Strength and Threshold of NOTCH Oncogenic Signals by mir-181a-1/b-1
Source: PLoS Genet. 2012 Aug 9;8(8):e1002855. doi: 10.1371/journal.pgen.1002855 (PMC3415433; doi:10.1371/journal.pgen.1002855)
Supplement: Figure S7 — Predicted base pairings between human Nrarp and miR-181a. (PDF) [file pgen.1002855.s007.pdf]

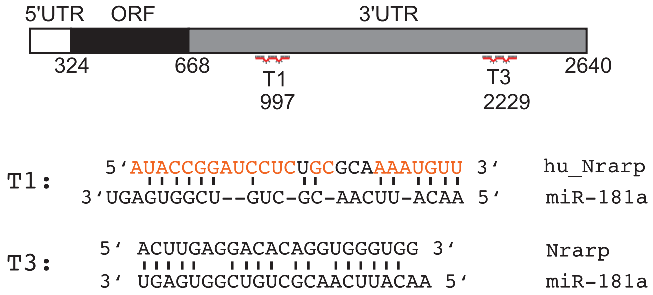
5'UTR
ORF
3'UTR
324
668
T1
997
T3
2229
2640
T1:
5' AUACCGGAUCCUCUGCGCAAAAUGUU 3' hu_Nrarp
3' UGAGUGGCU--GUC-GC-AACUU-ACAA 5' miR-181a
T3:
5' ACUUGAGGACACAGGUGGGUGG 3' Nrarp
3' UGAGUGGCUGUCGCAACUUACAA 5' miR-181a
