# Supplementary material for: Modulating the Strength and Threshold of NOTCH Oncogenic Signals by mir-181a-1/b-1
Source: PLoS Genet. 2012 Aug 9;8(8):e1002855. doi: 10.1371/journal.pgen.1002855 (PMC3415433; doi:10.1371/journal.pgen.1002855)
Supplement: Text S1 — Supplemental results and supplemental experimental procedures. (DOC) [file pgen.1002855.s012.doc]

**Modulating the Strength and Threshold of NOTCH Oncogenic Signals by *mir-181a-1/b-1***

Rita Fragoso ^1,2, #^, Tin Mao^1,2,#^, Song Wang ^1,2^, Steven Schaffert ^1,2^, Xue Gong^1,2^, Sibiao Yue ^1,2^, Richard Luong ^3^, Hyeyoung Min^1,2, 4^, Yumi Yashiro-Ohtani^5, 6^, Mark Davis ^7^, Warren Pear ^5, 6^ and Chang-Zheng Chen^1,2, *^

^1^ Department of Microbiology and Immunology, ^2^ Baxter Laboratory for Stem Cell Biology, ^3^ Department of Comparative Medicine, Stanford University School of Medicine, Stanford, CA 94305, USA; ^4^ Chung-Ang University College of Pharmacy, Seoul 156-756, Korea; ^5^ Abramson Family Cancer Research Institute and ^6^ Department of Pathology and Laboratory Medicine, University of Pennsylvania School of Medicine, Philadelphia, PA 19104, USA; ^7^  Howard Hughes Medical Institute, and Department of Microbiology and Immunology, Stanford University School of Medicine, Stanford, CA 94305, USA

^#^ These authors contributed equally to this work

* To whom correspondence should be addressed:

E-Mail: [czchen@stanford.edu](mailto:czchen@stanford.edu)

Telephone: 650-725-1667

**Supplemental Results**

**Pleotrophic effects of loss of *mir-181ab1* on ICN1-induced T-ALL**

In mice reconstituted with ICN1:*181ab1*^+/+^ BM cells, the percentage of GFP^+^ and GFP^+^DP cells in PB continuously increased at 2, 4 and 6 weeks after transplantation (Figure 3C and Figure S3A), whereas in mice reconstituted with ICN1:*181ab1*^–/–^ BM cells the percentage of GFP^+^ and GFP^+^DP cells first decreased from 2 to 4 weeks and then increased from 4 to 8 weeks post-transplantation (Figure 3C and Figure S3A). Similar alterations in the development of GFP^+^ and GFP^+^DP cells as a result of loss of *mir-181ab1* were observed in other hematopoietic/lymphoid organs, such as BM, spleen and thymus (Figure 3D-F), and in non-hematopoietic organs such as lungs and liver (Figure S3B-D). Of note, loss of *mir-181ab1* resulted in the development/migration of ICN1-infected DP cells in thymus at 2 weeks post-transplantation (Figure 3F, lower panel), suggesting that *mir-181ab1* can also modulate the tissue distribution/development of ICN1-infected T-ALL cells. Overall, FACS and histologic analyses of lymphoid organs of T-ALL mice indicated that loss of *mir-181ab1* had diverse effects during T-ALL development.

FACS and histologic analysis of lymphoid organs of T-cell acute lymphoblastic leukemia (T-ALL) mice confirmed that loss of *mir-181ab1* had diverse effects on the onset and development of T-ALL induced by ICN1 (Figure S3H). The thymi from both types of T-ALL mice appeared essentially normal with good delineation between the cortex and medulla at both 2- and 4-weeks of age. However, at 2 and 4 weeks post-transplantation, loss of *mir-181ab1* caused significant reduction in lymphocytic infiltration in T-ALL mice reconstituted with INC1-infected T-ALL cells. In T-ALL mice reconstituted with ICN1-infected *mir-181ab1* WT BM cells the femoral BM at 2- and 4-weeks post-transplantation was diffusely infiltrated by a uniform population of well-differentiated neoplastic lymphocytes (compatible with chronic lymphocytic leukemia). The white pulp of the spleen was hyperplastic at 2 weeks, but diffusely infiltrated by a uniform population of neoplastic lymphocytes at 4 weeks (compatible with splenic lymphoma/chronic lymphocytic leukemia). In contrast, in T-ALL mice reconstituted with ICN1-infected *mir-181ab1* knock-out BM cells, the femoral BM was not reconstituted adequately at 2 weeks post-transplantation, appearing hypocellular (predominantly consisting of white adipose tissue with only few scattered island of myeloid hematopoietic precursor cells). At 4-weeks post-transplantation, the BM appeared to have normal cellularity and identifiable erythroid hematopoietic precursor cells, myeloid hematopoietic precursor cells, and megakaryocytes display synchronous maturation. No infiltrated neoplastic lymphocytes were observed at 2 or 4 weeks post-transplantation. Similarly, the white pulp of the spleen was depleted, with virtual absence of lymphocytes that reveal the underlying dendritic and reticular architecture of the white pulp. These observations are consistent with the results of FACS-based analyses (Figure 3D-F), further supporting the role of *mir-181ab1* in ICN1-induced T-ALL.

Loss of *mir-181ab1* also compromised other steps that may be required for the development of ICN1-induced T-ALL. For example, loss of *mir-181ab1* enabled the appearance of ICN1-infected double-positive T cells (DP cells) at 2- and 4-weeks post-transplantation in thymus (Figure 3F). This is significant because ICN1 expression in hematopoietic progenitor cells is known to block T cell development in thymus while promoting ectopic T-cell development in BM and other organs. Furthermore, loss of *mir-181ab1* also attenuated ectopic T cell development in BM and spleen at 4 weeks after transplantation (Figure 3D, 3E). Finally, while loss of *mir-181ab1* did not have drastic effects on the development of ICN1-infected CD4 and CD8 double-negative (DN) subsets in thymus, it had varied effects on the development of DN subsets in BM (data not shown). These findings revealed the diverse effects of *mir-181ab1* in modulating the development of ICN1-infected T-ALL cells at various stages of T cell development in multiple tissues.

**The role of *mir-181ab1* in T-ALL maintenance**

To examine the role of *mir-181ab1* in T-ALL maintenance, we conditionally deleted *mir-181ab1* in mice transplanted with ICN1-infected *mir-181ab1^f/f^Cre-ER* BM cells at 2 weeks post-transplantation (Figure S8A, B). We found that induced deletion of *mir-181ab1* caused a significant decrease in DP leukemia cells with low levels of ICN1 expression (GFP^low^ and GFP^low^ DP cells) in the PB of recipient (Figure 8H). Furthermore, the percentages of GFP^low^ and GFP^low^ DP cells were maintained at lower levels throughout the assay after *mir-181ab1* deletion. In contrast, in mice with wild-type *mir-181ab1* alleles, the percentage of GFP^low^ cells increased with leukemia progression. These results suggest that the *mir-181ab1* gene or its products may be targeted for treatment of T-ALL maintained by weak Notch mutants with lower signaling strength.

**Supplemental Experimental Procedures**

**Generation of mir-181 knockout mice**

A genomic fragment containing the *mir-181ab*, or *mir-181ab2*, or *mir-181cd* cluster was excised out of a 129sv-derived BAC clone with the appropriate restriction enzymes to assemble the targeting constructs. The PGK-neo positive selection cassette flanked by FRT sites was subcloned from the pK11 plasmid (a gift from G. Martin) and the DT-A negative selection cassette was subcloned from the pMC1DTpA plasmid (a gift from T. Yagi). LoxP sites were placed at least 125 base pairs away from the miR-181a and miR-181b. Linearized targeting constructs were electroporated into CGR8 feeder-independent embryonic stem (ES) cells and G418-resistant clones with homologous recombination events were identified by PCR and confirmed by Southern blot analyses (data not shown). These clones were then expanded and injected into blastocysts to generate chimeras. Germline transmission for the *mir-181* floxed chimeras was confirmed by Southern blot and PCR analyses.

The conditional *mir-181* strains were first crossed with FLPeR [1] to remove the drug selection cassette and then crossed to Hprt-Cre [2] or CreER [3] mice for germline or inducible deletion of the *mir-181* alleles, respectively. Mouse strains are maintained on either a 129 background or mixed B6. *In vivo* conditional deletion of *mir-181ab1* during T-ALL development was induced by intraperitoneal injection of tamoxifen (2 mg/kg every 2 days for a 10-day period) into ICN1:*mir-181ab1^+/+^/CreER* or ICN*1:mir181a1b1^f/f^/CreER* T-ALL mice at 2 weeks after transplantation Figure S8B). *mir-181ab1* deletion was confirmed by PCR analysis of hematopoietic/lymphoid organs (Figure S8C).

**Plasmids**

We used retroviral expression vectors containing a murine stem cell virus backbone for the ectopic expression of miRNAs and/or target genes. miR-181a*^mut^* was generated by altering nucleotides 2 through 7 from the 5’ end of the mature miR-181a. Compensatory mutations were introduced into the miR-181a* strand to preserve the secondary structure of pre-miR181a. For Notch target gene over-expression, the full-length or mutant target genes were cloned downstream of mouse P_PGK_ and expressed as a bicistronic message with GFP placed after the EMCV internal ribosomal entry site (IRES) in the MDH1-PGK-IRES-GFP vector [4]. Retroviral constructs for expressing NOTCH1 oncogenes, MigR1, MigR1-ICN1, and MigR1-P12∆P, were previously described [5,6]. To co-express ICN1 and *mir-181a-1*, an *mir-181a-1* expression cassette driven by the human H1 promoter was inserted into the 3’ LTR of the MigR1-ICN1 viral vector [7]. The Nrarp open reading frame (ORF) was PCR-amplified from a full-length Mammalian Genome Collection (MGC) Clone (IMAGE 5391567; Accession BC069891; nt 107-451). Overexpression constructs for SHP-2, DUSP5, DUSP6 and PTPN22 were previously described [8].

**OP9-DL1 assay for *in vitro* thymocyte differentiation**

OP9-DL1 co-culture assays were used to examine the role of miRNAs in thymocyte development [4,9]. This protocol was modified such that total thymocytes were used to initiate the OP9-DL1 assay. Briefly, total thymocytes were isolated from 5-fluorouracil (5-FU) primed mice, infected with miRNA expressing viruses, and seeded onto OP9-DL1 stromal cells in culture medium with a cocktail of growth factors (5 ng/ml IL-7 and 27.5 ng/ml Flt3-Ligand). Thymocytes (100,000 cells) were seeded in each well of a 24-well plate containing a confluent monolayer of OP9-DL1 stromal cells. Twelve culture replicates were performed for each individual miRNA construct. At 24 hours after culture initiation, differentiated and non-adherent cells were removed by medium exchange. Cultures were fed with fresh medium at day 6 and analyzed at day 8 by FACS to determine the effects of miRNAs on CD4 and/or CD8 thymocyte development. Anti-CD45 antibody staining and/or FSC/SSC gating were used to differentiate infected thymocytes from GFP^+^ stromal cells. The results were summarized in box-plots to describe the %DP cells from individual cultures. In some cases, the results were normalized so that the negative control (empty vector) has a median activity of “0” and the wild-type *mir-181a-1* expressing vector had a median activity of “1.” The ends of the boxes define the 25^th^ and 75^th^ percentiles, a line indicates the median, and bars define the 5^th^ and 95^th^ percentiles. Such normalization is necessary to reset the baseline and allows for comparison among the independent repeats due to heterogeneous nature of the thymic progenitor cells and intrinsic variation between the batches of mice used in different experiments. Mann-Whitney rank sum tests were performed to determine the statistical significance.

**T-ALL cell lines**

To validate repression of Nrarp by miR-181a via western blot analyses, the Nrarp protein was tagged with two copies of HA (a.a. YPYDVPDYA) on the amino terminus and separated by amino acids GGS. The HA-Nrarp-FL was inserted downstream to P_PGK_ in the MDH1-PGK-IRES-GFP and transcribed as a bicistronic message along with GFP. MiR-181a or its mutant form was simultaneously expressed from the H1 expression cassette on the same vector. These co-expression constructs were packaged into retroviral viruses and used to infect T6E cell lines (kindly provided by Warren S. Pear, University of Pennsylvania). GFP^+^ cells were FACS-sorted to establish a stable cell line that was used for western blot analysis. Mouse T-ALL cell line T6E and human T-ALL cell line DND41 [10] were treated with antagomir against miR-181 as previous described [8].

**Fluorescence-activated cell sorting and analysis**

Total bone marrow cells were obtained by flushing off tibias and femurs, whereas single-cell suspensions of the spleen, thymus, lymph nodes, lungs and liver were obtained by grinding the tissues. After erythrocyte lysis, cells were stained with mouse anti-CD4 and anti-CD8 antibodies (eBioscience). To determine ICN1 expression levels, cells were first fixed and permeabilized, and then stained with a mouse anti-Notch1 antibody (BioLegend). Flow cytometry analysis was performed with a FACS Calibur and cell sorting was performed using a FACS Aria (both from BD).

**Antibodies for FACS and western blot analyses**

For FACS analysis, the following fluorescent-conjugated antibodies were obtained from eBioscience (San Diego, CA, USA): a-B220-FITC (RA3-6B2), a-CD3e-FITC (145-2C11), a-CD11b-FITC (M1/70), a-Ter-119-FITC (Ter-119), a-CD4-FITC (RM4-5), a-CD25-PE (PC61.5), a-CD4-PerCP/Cy5.5 (RM4-5), a-CD8a-APC (53-6.7), a-Gamma-Delta-TCR-FITC (UC7-13D5), a-CD8a-FITC (53-6.7), a-CD27-PE (LG-7F9), and a-CD3e-PE (145-2C11). The following antibody reagents were from BD (Franklin Lakes, NJ): a-B220-APC (RA3-6B2), a-B220-PE (RA3-6B2), a-BP-1-FITC (6C3), a-IgM-APC (II/41), a-IgD-FITC (11-26c.2a), a-CD5-APC (53-7.3), a-CD21-FITC (7G6), a-FAS-FITC (Jo2), a-CD19-APC (1D3), a-Gr-1-FITC (RB6-8C5), and a-TCRb-PE/Cy5 (H57-597). The following antibody reagents were from BioLegend (San Diego, CA): a-CD44-PerCP/Cy5.5 (IM7), a-CD69-APC (H1.2F3), a-CD62L-APC (MEL-14), a-CD11c-FITC (N418), a-NK1.1-FITC (PK136), a-cKit APC (2B8), a-CD25 PE/Cy7 (PC61) and a-CD43-PE-Cy5.5 (1B11). a-CD24-PE (30-F1) was obtained from Abcam (Cambridge, MA, USA), a-CD23-PE (2G8) was obtained from SouthernBiotech (Birmingham, AL, USA), and Peanut Agglutinin(PNA)-biotin was obtained from Vector Laboratories (Burlingame, CA, USA). For western blot analysis, the following antibodies were used: a-HA rabbit monoclonal antibody (DW2; Upstate), a-rabbit IgG (H+L)-HRP goat polyclonal antibody (Zymed Laboratories), a-Nrarp rabbit polyclonal antibody (Abcam), a-Dusp6 rabbit monoclonal antibody (Abcam), a-actin rabbit polyclonal antibody (Abcam), donkey a-rabbit IgG – HRP conjugated (Amershan Biosciences), and a-mouse b-actin rabbit polyclonal antibody (AbMart).

**Transfection of BOSC23 cells**

The BOSC23 cell line was used for transient transfection to generate retrovirus and for subsequent northern blot and western blot analyses. In each case, 5 X 10^5^ BOSC23 cells were plated on each well of a 6-well plate 12 h prior to transfection using FuGENE reagent (Roche Applied Science). For retroviral packaging, 2 mg of expression constructs (miRNA and/or target gene) were co-transfected with 1 mg pCLeco packaging vector. Retrovirus-containing supernatant was harvested at 48 h and immediately stored at -80°C in 1-ml aliquots. BOSC23 cells were lysed in TRIzol Reagent (Invitrogen) for northern blot analyses as previously described in detail [11]. To validate the shRNA-mediated knockdown of Nrarp protein expression, 2 mg of shRNA targeting the *Nrarp* gene (Figure 7E) was co-transfected with 1.25 mg of HA-Nrarp-FL expression construct. Cell lysate was harvested after 48 h and analyzed for Nrarp protein expression by western blot.

**RNA extraction and RT-qPCR analyses**

Total RNA was isolated using Trizol Reagent (Invitrogen) according to the manufacturer’s instructions. Absolute quantification of mature miR-181 family members was done using TaqMan miRNA assays (Applied Biosystems) as previously described [9]. Relative expression levels of target mRNAs were determined using TaqMan gene expression assays (Applied Biosystems). GAPDH was used as an internal control.

**Histology**

Tissues were fixed in a 10% buffered formalin solution for a minimum of 24 hours, embedded in paraffin, sectioned and stained with hematoxylin and eosin (HE). Before paraffin embedding, bones were decalcified using an EDTA solution.

**Mouse genotyping**

Southern probe templates were generated with genomic PCR with the specific primer sets.

(1) Primers for generating *mir-181a-1/b-1* Southern probe:

5’ AGGATGTAGGGATGTGAGGA 3’

5’ GTGTTCAGGACAGCAAAGAG 3’

(2) Primers for generating *mir-181a-2/b-2* Southern probe:

5’ CTAAGAATTCAAGCATATTCCTCTGTATCA 3’

5’ CTAAGAATTCTTAAGGTAGGAATTATAGGT 3’

(3) Primers for generating *mir-181c/d* Southern probe:

5’ TCAGTCTGCATCTGGCTACT 3’

5’ TCCTGCCTACCTTCTTCACT 3’

PCR analyses were performed on mouse tail DNA to confirm excision of the neo cassette and the deletion of the *mir-181a-1/b-1*, *mir-181a-2/b-2*, and *mir-181c/d* genes.

The primers used to amplify the *pgk-neo* cassette were:

5’ tgatgccgccgtgttcc 3’

5’ ggccacagtcgatgaatcca 3’

The *wild-type, floxed*, and *null* alleles of each *mir-181* gene were distinguished by 3-primer PCR.

(1) Primers for the *mir-181a-1/b-1* locus:

5’ TAACTTGAGAAAACCTAAGTGG 3’

5’ GGGAGCTTAGTTACAGGCTG 3’

5’ TCCCTGGAATCAAGTCTAC 3’

PCR products of 190 bp, 244 bp, and 306 bp resulted from *wild-type, floxed*, and *null* alleles, respectively.

(2) Primers for the *mir-181a-2/b-2* locus:

5’ GTGTTCAAGTTCTGGCTATG 3’

5’ GAGCTGCAGGATTGTGAGA 3’

5’ AAGGCATATTAATCGAATTC 3’

PCR products of 259 bp, 312 bp, and 218 bp resulted from *wild-type, floxed*, and *null* alleles, respectively.

(2) Primers for the *mir-181c/d* locus:

5’ TTTAGGTCAGGTTAGCCTGTA 3’

5’ AGACTCAGAAGAAGCAGCAC 3’

5’ TCATTCCCGCTCATCCATCC 3’

PCR products of 190 bp, 239 bp and 307 bp resulted from *wild-type, floxed*, and *null* alleles, respectively.

**Microarray expression profiling and miRNA target analyses**

Total RNAs were labeled using Illumina Total Prep RNA Amplification Kit and hybridized to Illumina MouseRef-8_V2 BEAD CHIP according manufacturer’s instruction. Data were normalized using the quantile method (the Bioconductor *lumi* package). SAM analyses were performed to select differentially expressed genes (> 2-fold, *p* < 0.01). Gene expression patterns were determined by hierarchical clustering with Pearson correlation as similarity metric. Heatmaps were generated with Gplots from the R package. Ingenuity Pathways Analyses (Ingenuity Systems, www.ingenuity.com) were carried out to determine the functional pathways with gene clusters. Gene set enrichment analyses (GSEA) were used to determine the effects of *mir-181ab1* deletion on the gene sets up- or down-regulated by ICN1 in T-ALL DP cells (Permutation=1000). miR-181a targets with perfect “seed” matches were identified using TargetScans 5.1 (<http://www.targetscan.org/>), PicTar (<http://pictar.mdc-berlin.de/>), or miRanda (<http://www.microrna.org/microrna/home.do>). Alternatively, m-fold program was used to identify putative miR-181a binding sites on selected target mRNAs. Sylamer analyses were carried out to observe the enrichment of seed matches among the genes up- or down-regulated in the absence of *mir-181ab1*. Control seeds were from other microRNAs in the miRBase (release 12).

**References**

1. Farley FW, Soriano P, Steffen LS, Dymecki SM (2000) Widespread recombinase expression using FLPeR (flipper) mice. Genesis 28: 106-110.

2. Tang SH, Silva FJ, Tsark WM, Mann JR (2002) A Cre/loxP-deleter transgenic line in mouse strain 129S1/SvImJ. Genesis 32: 199-202.

3. Ventura A, Kirsch DG, McLaughlin ME, Tuveson DA, Grimm J, et al. (2007) Restoration of p53 function leads to tumour regression in vivo. Nature 445: 661-665.

4. Mao TK, Chen CZ (2007) Dissecting microRNA-mediated gene regulation and function in T-cell development. Methods Enzymol 427: 171-189.

5. Pui JC, Allman D, Xu L, DeRocco S, Karnell FG, et al. (1999) Notch1 expression in early lymphopoiesis influences B versus T lineage determination. Immunity 11: 299-308.

6. Weng AP, Ferrando AA, Lee W, Morris JPt, Silverman LB, et al. (2004) Activating mutations of NOTCH1 in human T cell acute lymphoblastic leukemia. Science 306: 269-271.

7. Chen CZ, Li L, Lodish HF, Bartel DP (2004) MicroRNAs modulate hematopoietic lineage differentiation. Science 303: 83-86.

8. Li QJ, Chau J, Ebert PJ, Sylvester G, Min H, et al. (2007) miR-181a Is an Intrinsic Modulator of T Cell Sensitivity and Selection. Cell 129: 147-161.

9. Liu G, Min H, Yue S, Chen CZ (2008) Pre-miRNA loop nucleotides control the distinct activities of mir-181a-1 and mir-181c in early T cell development. PLoS ONE 3: e3592.

10. Hochstenbach F, Brenner MB (1989) T-cell receptor delta-chain can substitute for alpha to form a beta delta heterodimer. Nature 340: 562-565.

11. Min H, Chen CZ (2006) Methods for analyzing microRNA expression and function during hematopoietic lineage differentiation. Methods Mol Biol 342: 209-227.
